# Genomics meets metabolomics: decoding *Arnebia tschimganica* and the shikonin biosynthesis pathway

**DOI:** 10.1093/hr/uhag077

**Published:** 2026-03-03

**Authors:** Xuan Wang, Changyi Wang, Minkai Yang, Xiaohui Lai, Yile Sun, Tongming Yin, Bao Liu, Hansong Dong, Xiaobo Li, Zhonghao Ruan, Ju Huang, Aliya Fazal, Wencai Jie, Liu Yang, Xiaoran Lv, Hongwei Han, Dijun Chen, Guihua Lu, Sihai Yang, Zhongling Wen, Jinliang Qi, Yonghua Yang

**Affiliations:** State Key Laboratory of Pharmaceutical Biotechnology, Institute for Plant Molecular Biology, School of Life Sciences, Nanjing University, Nanjing 210023, China; Co-Innovation Center for Sustainable Forestry in Southern China, State Key Laboratory for Tree Genetics and Breeding, Nanjing Forestry University, Nanjing 210037, China; Xi’an Botanical Garden of Shaanxi Province, Institute of Botany of Shaanxi Province, No. 17 Cuihua South Road, Xi’an 710061, China; State Key Laboratory of Pharmaceutical Biotechnology, Institute for Plant Molecular Biology, School of Life Sciences, Nanjing University, Nanjing 210023, China; Co-Innovation Center for Sustainable Forestry in Southern China, State Key Laboratory for Tree Genetics and Breeding, Nanjing Forestry University, Nanjing 210037, China; State Key Laboratory of Pharmaceutical Biotechnology, Institute for Plant Molecular Biology, School of Life Sciences, Nanjing University, Nanjing 210023, China; Co-Innovation Center for Sustainable Forestry in Southern China, State Key Laboratory for Tree Genetics and Breeding, Nanjing Forestry University, Nanjing 210037, China; State Key Laboratory of Pharmaceutical Biotechnology, Institute for Plant Molecular Biology, School of Life Sciences, Nanjing University, Nanjing 210023, China; Co-Innovation Center for Sustainable Forestry in Southern China, State Key Laboratory for Tree Genetics and Breeding, Nanjing Forestry University, Nanjing 210037, China; State Key Laboratory of Pharmaceutical Biotechnology, Institute for Plant Molecular Biology, School of Life Sciences, Nanjing University, Nanjing 210023, China; Co-Innovation Center for Sustainable Forestry in Southern China, State Key Laboratory for Tree Genetics and Breeding, Nanjing Forestry University, Nanjing 210037, China; Key Laboratory of Molecular Epigenetics of the Ministry of Education (MOE), Northeast Normal University, Changchun 130024, China; College of Plant Protection, State Key Laboratory of Crop Biology, Shandong Agricultural University, Taian 271018, China; Technology Service Center, Annoroad Gene Technology (Beijing) Co., Ltd, Beijing 100176, China; State Key Laboratory of Pharmaceutical Biotechnology, Institute for Plant Molecular Biology, School of Life Sciences, Nanjing University, Nanjing 210023, China; State Key Laboratory of Pharmaceutical Biotechnology, Institute for Plant Molecular Biology, School of Life Sciences, Nanjing University, Nanjing 210023, China; State Key Laboratory of Pharmaceutical Biotechnology, Institute for Plant Molecular Biology, School of Life Sciences, Nanjing University, Nanjing 210023, China; Co-Innovation Center for Sustainable Forestry in Southern China, State Key Laboratory for Tree Genetics and Breeding, Nanjing Forestry University, Nanjing 210037, China; State Key Laboratory of Pharmaceutical Biotechnology, Institute for Plant Molecular Biology, School of Life Sciences, Nanjing University, Nanjing 210023, China; State Key Laboratory of Pharmaceutical Biotechnology, Institute for Plant Molecular Biology, School of Life Sciences, Nanjing University, Nanjing 210023, China; State Key Laboratory of Pharmaceutical Biotechnology, Institute for Plant Molecular Biology, School of Life Sciences, Nanjing University, Nanjing 210023, China; State Key Laboratory of Pharmaceutical Biotechnology, Institute for Plant Molecular Biology, School of Life Sciences, Nanjing University, Nanjing 210023, China; State Key Laboratory of Pharmaceutical Biotechnology, Institute for Plant Molecular Biology, School of Life Sciences, Nanjing University, Nanjing 210023, China; State Key Laboratory of Pharmaceutical Biotechnology, Institute for Plant Molecular Biology, School of Life Sciences, Nanjing University, Nanjing 210023, China; State Key Laboratory of Pharmaceutical Biotechnology, Institute for Plant Molecular Biology, School of Life Sciences, Nanjing University, Nanjing 210023, China; State Key Laboratory of Pharmaceutical Biotechnology, Institute for Plant Molecular Biology, School of Life Sciences, Nanjing University, Nanjing 210023, China; Co-Innovation Center for Sustainable Forestry in Southern China, State Key Laboratory for Tree Genetics and Breeding, Nanjing Forestry University, Nanjing 210037, China; State Key Laboratory of Pharmaceutical Biotechnology, Institute for Plant Molecular Biology, School of Life Sciences, Nanjing University, Nanjing 210023, China; Co-Innovation Center for Sustainable Forestry in Southern China, State Key Laboratory for Tree Genetics and Breeding, Nanjing Forestry University, Nanjing 210037, China; State Key Laboratory of Pharmaceutical Biotechnology, Institute for Plant Molecular Biology, School of Life Sciences, Nanjing University, Nanjing 210023, China; Co-Innovation Center for Sustainable Forestry in Southern China, State Key Laboratory for Tree Genetics and Breeding, Nanjing Forestry University, Nanjing 210037, China

## Abstract

*Arnebia tschimganica* is a vulnerable species within the Boraginaceae (Boraginales), which has long been taxonomically debated due to inconsistent molecular and morphological characteristics. Shikonin and its derivatives, which are found in the roots of Boraginaceae species, possess significant pharmacological and industrial potential; however, the regulatory mechanisms underlying their biosynthesis are not yet fully comprehended. The lack of reference genomes for *Arnebia* species has hindered further research in these fields. Here, this study sequenced and assembled the chromosome-level genome of *A. tschimganica*, revealing that Boraginales is sister to Lamiales within the lamiids and suggesting that the taxonomic status of *A. tschimganica* should be regressed from *Arnebia* to *Lithospermum*. *Arnebia tschimganica* has undergone a recent whole-genome duplication that is shared with other Boraginaceae species, and this event has driven the evolution of shikonin biosynthesis. Multi-omics analysis revealed significant differences in shikonin production between *A. tschimganica* and *Lithospermum erythrorhizon*, attributing reduced shikonin productions in *A. tschimganica* to low transcript levels of key biosynthetic genes postdivergence. Furthermore, AtsDSH1, the enzyme responsible for catalyzing the hydroxylation of deoxyshikonin to shikonin in *A. tschimganica*, was identified and functionally characterized. Two ERF transcription factors were identified as conserved regulators of the dehydroshikonin hydroxylase gene *DSH1*, potentially regulating shikonin biosynthesis. These findings provide a chromosome-level genomic perspective to clarify the taxonomy of this controversial swing species and advance valuable insights for shikonin biosynthesis regulation.

## Introduction

**Figure 1:**
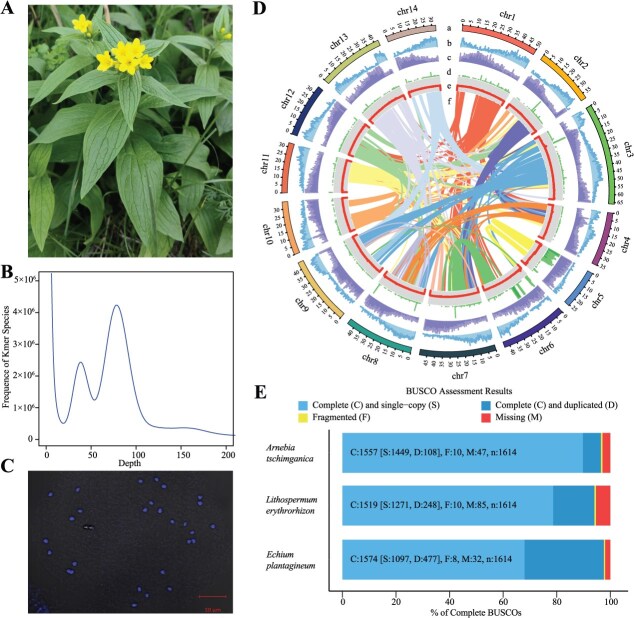
Morphological and assembled genomic features of *A. tschimganica*. (A) *Arnebia tschimganica* plant under natural growth conditions. (B) K-mer analysis for estimating the genome size of *A. tschimganica*. (C) Karyotype of *A. tschimganica*. Bar, 10 μm. (D) Distribution of *A. tschimganica* genomic features. (a) The pseudochromosomes, (b) gene density, (c) TEs density, (d) ncRNA density, (e) GC content, (f) intragenomic synteny. (E) Quality assessment of *A. tschimganica*, *L. erythrorhizon* and *E. plantagineum* genome assemblies using BUSCO tool based embryophyta_odb10 (number of genomes: 50, number of BUSCOs: 1614).

The Boraginaceae is a large group of plants consisting of 125 genera and over 2700 species [1]. It is widely distributed in temperate and tropical regions and has been an important source of medicinal plants in both traditional Chinese and Western medicine for centuries [2]. Among them, *Arnebia tschimganica* and *Lithospermum erythrorhizon* are traditional medicinal plants belonging to Boraginales–Boraginaceae–*Arnebia* and *Lithospermum*, respectively, in the Flora of China (Fig. 1A) [3]. Owing to its restricted population size, *A. tschimganica* is presently classified as vulnerable and urgently requires the protection of its genetic data and germplasm resources [4]. Shikonin and its derivatives are major chemical components extracted from roots of various plants within *Arnebia* and *Lithospermum* [5]. Contemporary investigations have demonstrated that shikonin and its derivatives exhibit a range of pharmacological properties, such as inhibiting foodborne pathogens [6], anti-SARS-CoV-2 [7], anti-inflammatory [8], suppression of tumor progression and metastasis [9, 10], as well as promoting wound healing [11]. Additionally, shikonin serves as a high-quality natural pigment with applications in cosmetics and dyeing industries. Comprehensive investigation into the composition and biosynthesis of shikonin in Boraginaceae roots is essential to enhance production and meet industrial demand for these natural products [12, 13].

The biosynthesis of shikonin and its derivatives involves two upstream pathways and one core downstream pathway. In the upstream pathways, geranyl pyrophosphate (GPP) is synthesized through the mevalonate (MVA) pathway [14], while the *p*-hydroxybenzoic acid (PHB) is produced through the phenylpropanoid (PP) pathway [15]. This is followed by downstream conversion into shikonin via several enzymatic reactions: *p*-hydroxybenzoate-geranyltransferase (PGT) catalyzed the synthesis of GPP and PHB into geranyl-4-hydroxybenzoate, which is subsequently converted to geranyl-hydroquinone (GHQ) [16, 17]; GHQ was then catalyzed by enzymes such as GHQ3″H and DSH to produce shikonin [18–20]; then shikonin was subsequently acetylated to acetylshikonin by BAHD [21]. The biosynthesis of shikonin and its derivatives is constrained by regulation of transcription factors (TFs). *LeERF-1* and *LeEIL-1* may be involved in the regulation of shikonin biosynthesis through light and ethylene signaling pathways, respectively [22, 23]. Overexpression of *LeMYB1* can significantly upregulate the synthesis of PAL, HMGR, and PGT, as well as induce the expression of *LeDI-2* and *LePS-2*, resulting in a 2.8-fold increase in shikonin production [24]. Additionally, five WRKY transcription factors have been predicted to be associated with shikonin biosynthesis [25]. Nevertheless, the transcription factors regulating the target enzyme genes involved in shikonin biosynthesis, and its regulatory mechanisms are still poorly understood, impeding efficient biosynthesis and development research of shikonin in plants. This remains to be resolved through molecular biology research based on multi-omics data.

The Boraginales consists of only Boraginaceae, which together with Lamiales, Gentianales, and Solanales forms the core group of the lamiids [26]. Currently, a considerable number of studies employing chloroplast genes such as atpB, rbcL, ndhF, rps16, trnL-trnF, and matK, nuclear markers of 18S nrDNA and 26S nrDNA, as well as genomic/transcriptomic data have resulted in 10 distinct topologies. This indicates that the systematic relationship among these orders has not been clarified, and the evolutionary position of Borageales within lamiids remains ambiguous [26–29]. In addition, the taxonomic status of *A. tschimganica* (=*Ulugbekia tschimganica* = *Lithospermum tschimganicum*) has been controversial since Johnston identified it as *Lithospermum* [30]. Then Zakirov created monotypic *Ulugbekia* for *L. tschimganicum*, who contended that *L. tschimganicum* exhibits morphological characteristics distinct from *Lithospermum*: corollas without faucal appendages and the insertion of the filaments on the anthers [31]. Nevertheless, Zhu *et al.* classified it into *Arnebia* based on the fact that it had morphological features consistent with those of *Arnebia* species: heterodistylous flower, the apex of the style is shallowly two-lobed, each with a two-lobed stigma, and blunt apex of anther [32]. This classification has been adopted by Plants of the World Online [33]. In the taxonomic studies based on molecular evidence, Weigend *et al.* constructed a phylogenetic tree using 5.8s rRNA, trnL–trnF, and trnS–trnG from multiple Boraginales species, showing that *A. tschimganica* should be classified within the monotypic genus *Ulugbekia*, which is closely related to *Lithospermum* but retains its taxonomic independence [34]. Hence, to better comprehend the systemic position of Boraginales and *A. tschimganica*, it is urgently necessary to supplement the genome information of more Boraginales species.

Here, we report the high-quality chromosome-level genome of *A. tschimganica* and supplement tissue transcriptome information from five Boraginales species. A comprehensive comparative analysis of the genome, metabolome, and transcriptome of *A. tschimganica* and *L. erythrorhizon* offers valuable insights into the taxonomic status of Boraginales and *A. tschimganica*, as well as the genetic basis underlying the differences of the important medicinal compounds—shikonin and its derivatives—among closely related species. Furthermore, we identified the dehydroshikonin hydroxylase gene *AtsDSH1* and two ERF transcription factors that activate its expression. These results provide an essential theoretical and molecular basis for the further investigation into the biosynthesis and regulatory mechanisms of shikonin and its derivatives.

## Results

### Genome assembly and annotation of *A. tschimganica*

Based on k-mer = 17 analysis of 76.32 Gb Illumina reads, the genome size of the diploid species *A. tschimganica* was estimated to be 582 Mb, with a heterozygosity of 0.87%. This is consistent with the estimated single-haploid genome size (0.59 Gb) obtained by flow cytometry (Fig. 1B, Fig. S1). Using 25.55 Gb (42×) of high-fidelity (HiFi) reads with an N50 length of 18 345 bp, primary contigs were assembled. Subsequently, 94.26% of contig sequences were anchored to 14 chromosomes using 60.51 Gb (100×) Hi-C clean reads (Fig. 1C, D, Fig. S2). The total genome sequence length of *A. tschimganica* was determined to be 611.54 Mb, consisting of 108 scaffolds with a scaffold N50 of 42.9 Mb (Table 1). Compared to the genome of *L. erythrorhizon* (693.34 Mb), the assembly continuity of *A. tschimganica* is superior, as evidenced by its contig N50 of 15.2 Mb, which is longer than that of *L. erythrorhizon* (~238.08 Kb) [35]. BUSCO analysis revealed that among the conserved core embryophyte genes, our assembly comprised 1557 (96.5%) complete embryophyte genes (1449 single-copy and 108 duplicated genes), which significantly surpassed the quality of the genome assemblies for *L. erythrorhizon* and *Echium plantagineum* within the Boraginaceae family (Fig. 1E). Furthermore, 99.2% of survey reads were mapped back to the assembly, confirming a genome coverage of up to 99.98%. These findings, combined with a high-quality LTR assembly index (LAI) value of 25.63, highlight the exceptional quality of the *A. tschimganica* genome assembly.

The *A. tschimganica* genome sequence comprises 370.28 Mb (60.55%) of repetitive sequences, predominantly long terminal repeat retrotransposons (LTR-RTs). Among them, LTR/Gypsy elements account for the largest proportion, constituting 21.39% of the genome sequence (Table S1). A total of 35 059 protein-coding genes were predicted, of which 31 640 are supported by transcriptome evidence. The average gene length is 3778.67 bp, while the average length of coding DNA sequences (CDS) is 1203 bp (Table S2). Functional annotations were assigned to 96.89% of the protein-coding genes in at least one database (Table S3). Additionally, the genome includes various noncoding RNA (ncRNA), comprising 17 649 rRNA, 1181 tRNA, 192 miRNA, and 777 snRNA (Table S4).

### Comparative genomics/transcriptomics analysis has reclassified the taxonomic status of *A. tschimganica*

**Figure 2:**
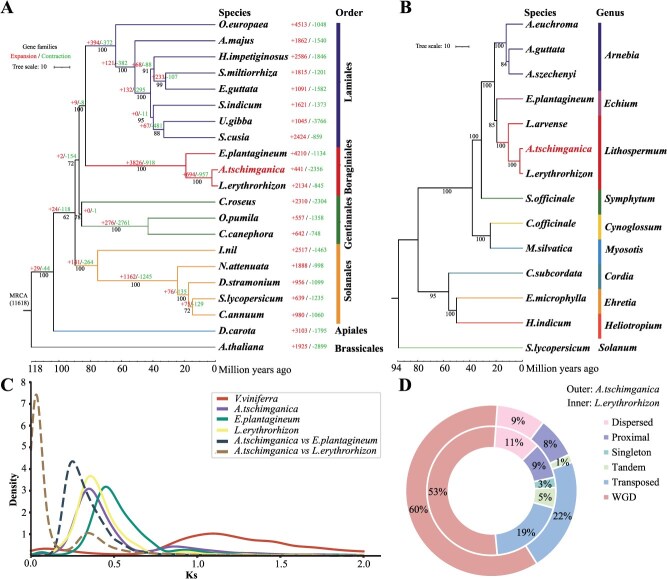
Comparative genomic/transcriptomics analysis of *A. tschimganica* and *L. erythrorhizon*. (A) The phylogenetic tree of Boraginales, Gentianales, Solanales, and Lamiales constructed using 130 single copy orthologs genes. (B) The phylogenetic tree of *A. tschimganica* constructed using 137 single-copy orthologous genes. (C) Synonymous substitution rates (*Ks*) distributions of syntenic blocks for the paralogous and orthologous of *A. tschimganica*, *L. erythrorhizon*, *E. plantagineum*, and *V. vinifera*. The solid lines represent Ks distributions for paralogous gene pairs derived from syntenic blocks within *V. vinifera* (red), *A. tschimganica* (purple), *E. plantagineum* (green), and *L. erythrorhizon* (yellow). The dashed lines represent Ks distributions for orthologous gene pairs derived from syntenic blocks between *A. tschimganica* and each of two related species: *L. erythrorhizon* (brown) and *E. plantagineum* (dark blue). (D) Statistics of duplication types of shikonin biosynthesis genes in *A. tschimganica* and *L. erythrorhizon*.

The sequencing and assembly of the *A. tschimganica* genome provides an opportunity to explore the phylogenetic evolution of both Boraginales and *A. tschimganica*. Using 130 single-copy orthologous genes from 21 representative species (3 Boraginales, 5 Solanales, 2 Gentianales, 8 Lamiales, and 3 outgroup species) (Table S5), we inferred the systematic evolutionary status of the Boraginales within the lamiid core clade (Boraginales, Solanales, Gentianales, and Lamiales). The topology of the phylogenetic tree suggests that Boraginales is most closely related to Lamiales, with an estimated divergence time of 83.3 million years ago (MYA). Gentianales forms a sister branch to the common ancestor of Boraginales and Lamiales, which diverged from Solanales (Fig. 2A). The phylogenetic tree constructed using syntenic orthogroups inferred with the Orthology Index (OI) = 0.6 also exhibited a consistent topology: [[Boraginales, Lamiales], Gentianales], Solanales] (Fig. S3).

**Table 1:** The assembly statistics of *A. tschimganica* genome.

**Feature**	**Value**
Raw data of HiFi reads (Gb)	25.55
Raw data of Hi-C reads (Gb)	60.51
Estimated genome size (Mb)	582
Assembled contigs (Mb)	611.54
Contig N50 (Mb)	15.20
Contig number	165
Assembled scaffolds (Mb)	611.54
Scaffold N50 (Mb)	42.90
Scaffold number	108
Pseudo-chromosomes	14
Anchored to chromosomes (Mb)	576.48
GC content (%)	36
Number of genes	35 059

In order to provide a more comprehensive analysis of the taxonomic status of *A. tschimganica*, we first supplemented transcriptomic data with unigenes from several Boraginaceae plants including *Arnebia guttata*, *Arnebia szechenyi*, *Lithospermum arvense*, *Cordia subcordata*, and *Heliotropium indicum*. Then the phylogenetic tree of 14 representative species in the Boraginaceae was constructed using 137 single-copy orthologous genes based on the maximum likelihood, with *Solanum lycopersicum* as the outgroup (Table S6). The result indicated that *A. tschimganica* was identified as a sister lineage to *L. erythrorhizon*, and is not clustered within the *Arnebia* branch, which includes *Arnebia euchroma*, *A. guttata*, and *A. szechenyi* (Fig. 2B). This topology received 100% bootstrap support. The phylogenetic tree constructed using syntenic orthogroups inferred with the OI = 0.6 also exhibited the consistent topology (Fig. S4). The divergence time between *Arnebia* and the ancestor of *Lithospermum* and *Echium* was estimated to have occurred ~21.4 MYA. Based on the current molecular evidence, we propose a new hypothesis that *A. tschimganica* should be regressed under *Lithospermum* rather than being classified within *Arnebia*. The three Boraginaceae species exhibit distinct patterns of gene family expansion and contraction (Fig. 2A). Compared with *L. erythrorhizon* and *E. plantagineum*, *A. tschimganica* exhibited the least number of gene families that underwent expansion and the highest number of gene families that experienced contraction. To understand the differential evolution and adaptation of *A. tschimganica*, Gene Ontology (GO) term enrichment analyses were performed on its 441 expanded and 2356 contracted gene families. The analysis revealed that a significant number of genes are involved in metabolic processes, biological regulation, and responses to stimuli, primarily associated with catalytic activity, transporter activity, binding, and transcription regulator activity (Fig. S5). This finding indicates that *A. tschimganica* has reduced responsiveness to secondary metabolism and stress responses throughout its developmental stages.

### Shared WGD event contributed to shikonin biosynthesis in *A. tschimganica* and *L. erythrorhizon*

Whole-genome duplication (WGD) and transposon-mediated genomic rearrangements play pivotal roles in shaping plant evolution and developmental traits, occurring nearly in all land plant lineages. To explore the traces of WGD in Boraginaceae species, synonymous substitution rate (Ks) values were estimated based on paralogous gene pairs in syntenic regions of *A. tschimganica* and two other shikonin-producing species. The Ks frequency in *A. tschimganica*, *L. erythrorhizon*, and *E. plantagineum* showed two peaks at ~0.349–0.451 and 0.864–0.927. Although the timing of the minor peak at Ks = 0.864–0.927 differs from that of the ancient gamma event shared by the core eudicots, this signal could be influenced by variable substitution rates. Furthermore, the corresponding syntenic blocks supporting this peak are few and highly fragmented, lacking robust evidence for synteny. Therefore, we conclude that the three Boraginaceae species have undergone at least one shared, recent WGD event (Fig. 2C, Fig. S6). This finding was further supported by high-confidence syntenic orthologs obtained after applying a more stringent filtering with OI = 0.6 using the SOI (Fig. S6) [36].

A total of 31 315 duplicated genes in *A. tschimganica* and 33 286 duplicated genes in *L. erythrorhizon* were identified and classified into five categories: 7627 and 9056 dispersed duplication genes, 12 636 and 13 340 WGD genes, 7761 and 9472 transposed duplication genes, 1561 and 941 tandem duplication genes, and 1730 and 477 proximal duplication genes (Table S7). Next, we identified 65 and 78 enzyme genes involved in shikonin biosynthesis from the *A. tschimganica* and *L. erythrorhizon* genomes and investigated the duplications of these genes, including 22 and 28 genes in the MVA pathway, 18 and 22 genes in the PP pathway, and 25 and 28 genes in the downstream core pathway, respectively (Table S8). Notably, 60% of the shikonin biosynthesis genes in *A. tschimganica* and 53% in *L. erythrorhizon* were retained following WGD (Fig. 2D, Table S8). Furthermore, the median Ks values for most syntenic blocks containing WGD-related shikonin biosynthesis genes ranged from ~0.35 to 0.44, which corresponds closely to the first peak observed in the Ks distribution (Tables S9 and S10). These findings suggest that the recent shared WGD event in Bignoniaceae played an important role in the expansion of shikonin biosynthesis gene family and the evolutionary development of shikonin biosynthesis in shikonin-producing species.

### Comprehensive metabolic profiling and differential analysis of root metabolites in *A. tschimganica* and *L. erythrorhizon*

**Figure 3:**
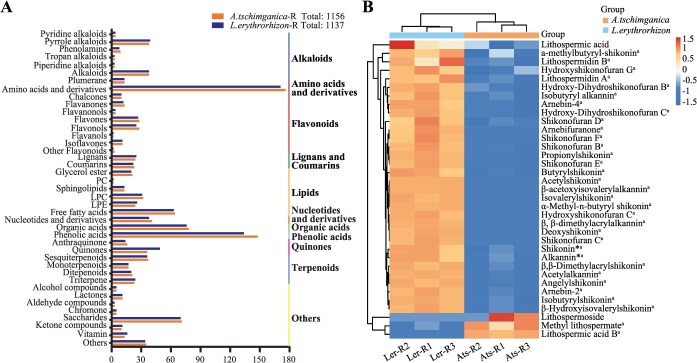
Widely targeted metabolomics analysis of roots extraction of *A. tschimganica* and *L. erythrorhizon*. (A) Composition of metabolites in the root of *A. tschimganica* and *L. erythrorhizon*. (B) Heatmap of relative content of shikonin and its derivatives in the roots of *A. tschimganica* and *L. erythrorhizon*. The label a in the top right corner indicates that the relative content of metabolite is significantly different between the two species. Ler-R: the root of *L. erythrorhizon*; Ats-R: the root of *A. tschimganica*.

Investigating the metabolic characteristics and species differences in the roots of closely related plants within the *Lithospermum* is essential for understanding the evolution of shikonin biosynthesis and evaluating its pharmaceutical potential. Using high-sensitivity, broad-coverage, and targeted metabolomics analysis techniques, we extracted metabolites from the roots of *A. tschimganica* and *L. erythrorhizon*. A total of 1156 and 1137 metabolites were identified, with comparable compositions that include 11 classes such as quinones, alkaloids, flavonoids, lipids, lignans and coumarins, phenolic acids, terpenoids, amino acids and derivatives, nucleotides and derivatives, organic acids, and others (Fig. 3A). The results show that *A. tschimganica* contains a greater variety of flavonoids, phenolic acids, and amino acid derivatives compared to *L. erythrorhizon*. Conversely, *L. erythrorhizon* exhibits greater diversity in quinones (Fig. 3A). Differential metabolite analysis reveals significant variations in the abundance of 644 metabolites between the two species, *A. tschimganica* and *L. erythrorhizon* (Fig. S7). In *A. tschimganica*, 309 metabolites were upregulated compared to *L. erythrorhizon*, with phenolic acids, amino acids and their derivatives, and lipids comprising the largest proportions. Conversely, 335 metabolites were downregulated, primarily quinones, amino acids and their derivatives, and alkaloids. Among these, deoxyshikonin exhibited the greatest magnitude of decrease, with a fold change of ~17.6 (Table S11). The top 3 enriched pathways for these differential metabolites are metabolic pathways, biosynthesis of secondary metabolites, and biosynthesis of amino acids (Fig. S8).

As characteristic metabolites of Boraginaceae plants, a total of 23 shikonin and its derivatives have been identified in *A. tschimganica* while *L. erythrorhizon* contains a total of 31 such compounds. Among them, the relative contents of 23 shikonin and its derivatives, such as deoxyshikonin, shikonin, acetylshikonin, and isobutylshikonin, were significantly lower in the roots of *A. tschimganica* compared to *L. erythrorhizon*. Additionally, lithospermic acid B and methyl lithospermate were significantly upregulated in *A. tschimganica* (Fig. 3B). These findings indicate significant disparities in the production of shikonin and its derivatives in the roots of these two closely related species following their divergence.

### The shikonin differences between close relative species are closely related to the transcription level of shikonin biosynthesis genes

**Figure 4:**
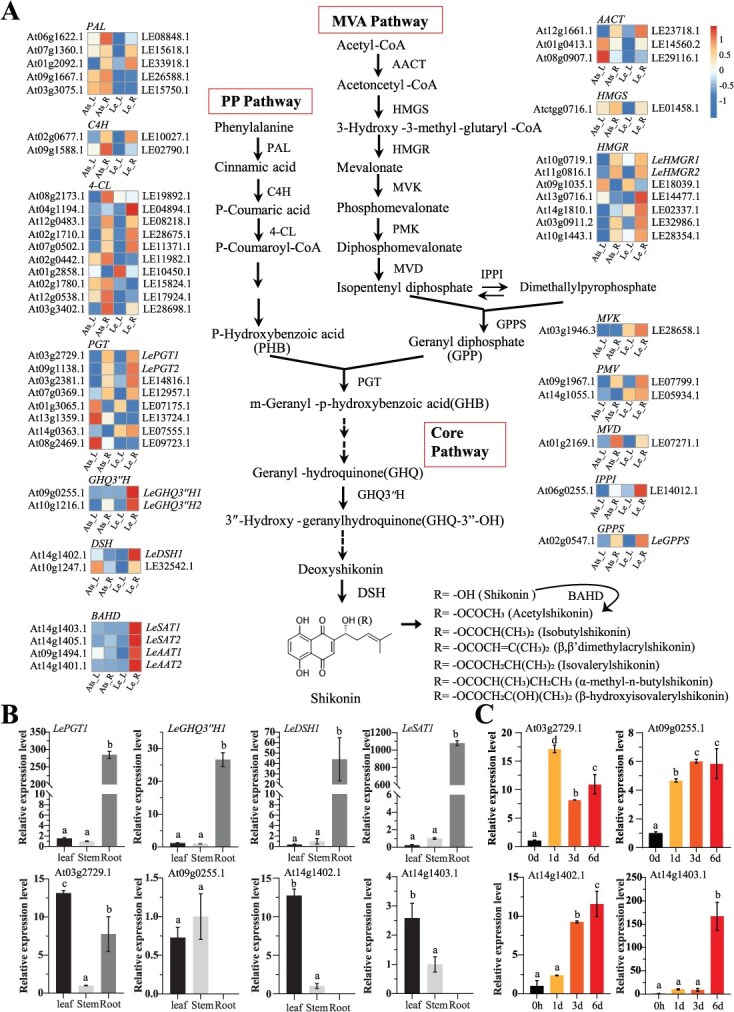
The cross-species comparative transcriptome analysis of shikonin and its derivatives biosynthesis genes in *A. tschimganica* and *L. erythrorhizon*. (A) The heatmap of the expression level of shikonin and its derivatives biosynthesis genes in *A. tschimganica* and *L. erythrorhizon*. The left and right sides of the heatmap frame are the corresponding orthologous genes in *A. tschimganica* and *L. erythrorhizon* respectively. (B) Tissue (leaf, stem and root) expression patterns of the core pathway genes of shikonin and its derivatives biosynthesis in *A. tschimganica* and *L. erythrorhizon*. (C) Relative expression levels of the core pathway genes for shikonin and its derivatives biosynthesis in *A. tschimganica* callus cells cultured in M9 and darkness for different times (0 h, 1 day, 3 days, and 6 days) were detected using qPCR. The error bars reflect the standard deviations of three biological replicates. a, b and c represent significant differences via the ordinary one-way ANOVA analysis.

Since the ortholog genes involved in shikonin biosynthesis have been identified in *A. tschimganica* and *L. erythrorhizon*, the reduction in shikonin and its derivative contents was root specific, with no detectable levels found in the stem or leaf of either species (Fig. S9). We performed a cross-species comparative transcriptome analysis using tissue transcriptome data to elucidate the potential reasons for the observed differences in shikonin and its derivative content in the roots of these two species following divergence. Most enzyme genes within MVA and PP pathways, as well as *PGT1*/*PGT2* and *GHQ3″H2*, exhibited high expression levels in both species’ roots, although their expression levels were consistently lower in *A. tschimganica* compared to those in *L. erythrorhizon*. Notably, genes such as *LeGHQ3″H1*, *LeDSH1*, and *LeSAT1* showed markedly high expression levels in *L. erythrorhizon* roots, while their orthologs in *A. tschimganica* exhibited extremely low expression levels, with TPM values below 10, indicating minimal or no expression (Fig. 4A). This is consistent with the extremely low levels of dehydroshikonin, shikonin, and acetylshikonin detected in *A. tschimganica* roots (Fig. 3B). The correlation heatmap analysis of gene expression levels of shikonin biosynthetic enzymes versus shikonin and its derivatives contents shows that the expression levels of genes such as *GHQ3″H1*, *DSH1*, and *SAT1* are significantly correlated with the contents of deoxyshikonin, shikonin, and its derivatives (Fig. S10). And quantitative PCR further validated the tissue expression patterns of four key genes in the downstream pathway across both species (Fig. 4B). These findings suggest that significant variations in shikonin and its derivatives between these closely related species are closely linked to transcript levels of enzyme genes involved in shikonin biosynthesis.

Subsequently, we induced callus cells from *A. tschimganica* and transferred them to M9 medium under dark culture conditions, which provides the specific conditions conducive to shikonin biosynthesis. As anticipated, the expression of core pathway genes was significantly upregulated with prolonged culture time (Fig. 4C). This upregulation facilitated the gradual synthesis of shikonin, acetylshikonin, and β-hydroxyisovalerylshikonin (Fig. S11). Notably, the expression levels of At09g0255.1 (*GHQ3″H1*), At14g1402.1 (*DSH1*), and At14g1403.1 (*SAT1*) increased by 6–150 times by day 6, despite being nearly undetectable in *A. tschimganica* roots (Fig. 4C). These results further reinforce the hypothesis that the extremely low transcript levels of core pathway genes are a key factor contributing to the reduced shikonin production in *A. tschimganica* after its divergence from its closely related species.

### AtsDSH1 catalyzes the hydroxylation of deoxyshikonin to shikonin

**Figure 5:**
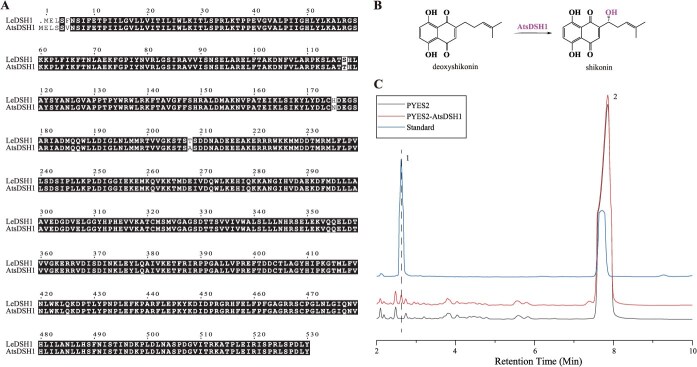
Functional characterization of AtsDSH1 for catalyzing deoxyshikonin to shikonin. (A) Sequence alignment of the amino acid of LeDSH1 and the corresponding orthologous AtsDSH1 (At14g1402.1) in *A. tschimganica*. (B) DSH1 catalyzes the synthesis of shikonin from deoxyshikonin. (C) HPLC-DAD analysis (detection at 515 nm) of extracts from the substrate-feeding experiment in yeast strain WAT11. Chromatograms are shown for the negative control (yeast carrying the empty vector pYES2; grey), the experimental group (yeast expressing pYES2::AtsDSH1; red), and standards of shikonin (1) and deoxyshikonin (2) (blue).

The oxidative hydroxylation catalyzed by DSH1 represents the final step in shikonin biosynthesis and plays a pivotal role. Given the 99.25% sequence similarity between At14g1402.1 and LeDSH1 (Fig. 5A), we concluded At14g1402.1 as deoxyshikonin hydroxylase AtsDSH1, which is responsible for catalyzing shikonin biosynthesis in *A. tschimganica* (Fig. 5B).

To further confirm the function of AtsDSH1 in catalyzing the hydroxylation of deoxyshikonin to produce shikonin, the full-length *AtsDSH1* gene was cloned into the yeast expression vector pYES2. The recombinant plasmid was then transformed into the yeast (*Saccharomyces cerevisiae*) strain WAT11, which co-expresses AtCPR1 from *Arabidopsis thaliana*, for subsequent functional characterization. A yeast strain carrying the empty pYES2 vector served as the negative control. The engineered yeasts were cultured and fed with 2 mg of deoxyshikonin in 7.5-ml SC-Ura cultures. After 48 h, methanol extracts of the harvested yeast cells were analyzed by HPLC. The results showed that the yeast strain expressing AtsDSH1 converted deoxyshikonin (substrate 2) into a new product (product 1), which shared an identical retention time (2.62 min) with the shikonin standard (Fig. 5C). In contrast, no corresponding peak was detected at this retention time in the extract from yeast harboring the empty pYES2 vector (Fig. 5C). These findings conclusively demonstrate that AtsDSH1 catalyzes the hydroxylation of deoxyshikonin to form shikonin.

### Two ERF transcription factors can activate *DSH1* to regulate biosynthesis of shikonin

**Figure 6:**
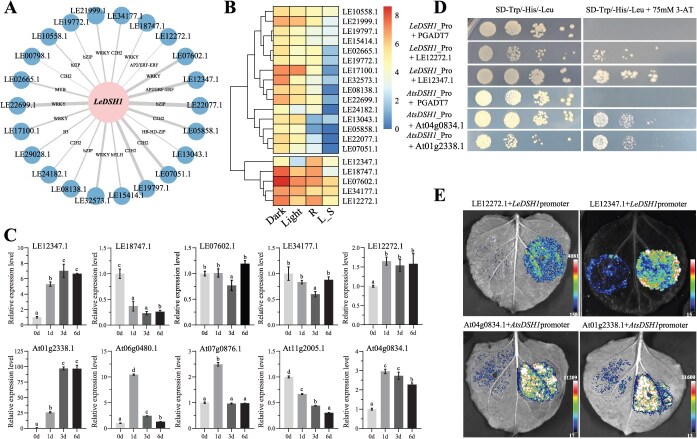
The ERF transcription factors activate the transcription of *DSH1* for shikonin biosynthesis. (A) Twenty-two candidate transcription factors of *LeDSH1* were screened through the co-expression network analysis. (B) Heatmap of tissue and callus culture expression patterns of candidate transcription factors. (C) The expression patterns of candidate transcription factors in M9 and dark culture callus were verified by qPCR. (D) The interaction between two candidate ERFs and *DSH1* promoter was verified by Y1H assays. (E) The dual luciferase reporter system verified the transcriptional activation of *DSH1* promoters by two candidate ERFs.

The high transcriptional levels of enzyme genes are crucial for shikonin biosynthesis, making it critical to understand the regulatory mechanisms governing their expression for the sustainable production of shikonin and its derivatives. To explore the transcription factors regulating the expression of *DSH1*, which encodes the key enzyme responsible for the final step in shikonin biosynthesis, we performed a co-expression network analysis involving *LeDSH1* and transcription factors using published tissue transcriptome data, identifying 22 candidate *LeTFs* that were highly co-expressed with *LeDSH1* (Fig. 6A). Among these, ERF transcription factors LE12347.1 and LE12272.1, WRKY transcription factor LE18747.1, and the C2H2 transcription factors LE07602.1 and LE34177.1 exhibited high expression levels in roots and during M9 dark culture (Fig. 6B).

Their orthologs in *A. tschimganica* were further identified, and their expression patterns were verified through qPCR analysis. Results indicated significant upregulation of LE12347.1 and LE12272.1 along with their orthologs (At01g2338.1 and At04g0834.1) during prolonged M9 dark culture, demonstrating consistent trends across both species (Fig. 6C). In contrast, LE18747.1, LE07602.1, and LE34177.1 displayed transcriptional downregulation under M9 dark culture conditions or inconsistent trends relative to their orthologs in *A. tschimganica* (Fig. 6C).

Additionally, cis-acting elements associated with ERF transcription factors—DRE/CRT and GCC box—were identified at −1689 bp and −1810 bp within the promoter region of *LeDSH1* and at −1049 bp and −1399 bp within the promoter region of *AtsDSH1* (Fig. S12). Consequently, LE12347.1 (LeERF2) and LE12272.1 (LeERF3), along with At01g2338.1 (AtsERF2) and At04g0834.1 (AtsERF3), were selected as candidate transcription factors to further investigate their binding to *DSH1’s* promoters. Yeast one-hybrid (Y1H) assays demonstrated that these factors could bind to the promoter regions of *LeDSH1* and *AtsDSH1* (Fig. 6D). Additionally, dual luciferase reporter assays confirmed their conserved transcriptional activation of both promoters across related species (Fig. 6E). These results suggest that these two ERF transcription factors play important regulatory roles in shikonin biosynthesis and are potential targets for gene engineering aimed at enhancing shikonin production. Through comparative transcriptomic analysis, we also identified six transcription factors (LE15414.1, LE18747.1, LE21999.1, LE22699.1, LE00798.1, LE19797.1) exhibiting significantly elevated expression in the roots of *L. erythrorhizon* relative to leaves. Their orthologs in *A. tschimganica* (At03g2449.1, At06g0480.1, At08g0103.1, At01g1696.1, At07g1220.1, At02g1265.1) showed no tissue-specific differential expression between roots and leaves. Notably, the root expression levels of At07g1220.1, At08g0103.1, and At06g0480.1 were substantially lower than those of their orthologs LE00798.1, LE21999.1, and LE18747.1, respectively (Fig. S13). We hypothesize that these transcriptional regulators may contribute to the interspecific divergence in shikonin biosynthesis between *A. tschimganica* and *L. erythrorhizon*.

## Discussion

**Figure 7:**
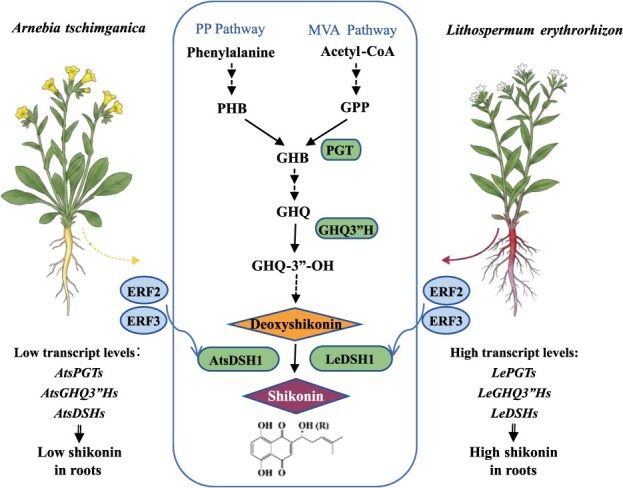
A schematic model illustrating the mechanisms underlying differences in shikonin content between *A. tschimganica* and *L. erythrorhizon*, and the role of the DSH1 enzyme gene and its transcriptional regulation in shikonin biosynthesis.

As representative plants of Boraginaceae, the roots of *A. tschimganica* and *L. erythrorhizon* can produce shikonin and its derivatives, which have well-known medicinal and industrial value. Here, we constructed a high-quality chromosome-level reference genome of *A. tschimganica*, the first of its kind within the *Arnebia* genus. Comparative genomic and transcriptomic analysis have provided new insights into the phylogenetic status of Boraginales and *A. tschimganica*, as well as the shared whole genome duplication (WGD) event in three Boraginales species related to shikonin biosynthesis. The low shikonin content in *A. tschimganica* provides a critical contrasting system to high-yielding species like *L. erythrorhizon*. By comparing their genomes and transcriptomes, we explored the causes of differences in shikonin biosynthesis content among closely related species, and also identified the key enzyme catalyzing the hydroxylation of deoxyshikonin to shikonin in *A. tschimganica*, as well as two ERF transcription factors involved in this process (Fig. 7). The genomics and metabolomics resources developed in this study will aid in effective germplasm conservation and utilization while providing an essential theoretical basis for further investigation into the biosynthesis and regulatory mechanisms of shikonin and its derivatives.

The phylogeny of the core group of lamiids and *A. tschimganica* has been a subject of long-term interest in research. High-quality genomes are the foundation for performing robust phylogenomic analyses. By integrating published genomes from the core group of lamiids, we propose that the phylogenetic relationships within this group are [[[Boraginales, Lamiales], Gentianales], Solanales], which align with the findings of Albach *et al.* [37]. Among the other findings based on omics data, Zhang *et al.* concluded that the lamiids are divided into two sister clades, clade I consisting of [Lamiales, Vahliales] and clade II composed of [[Boraginales, Gentianales], Solanales], based on 387 low-copy genes from 213 transcriptomes/genomes of Asterid species [28]. Additionally, Tang *et al.* and Auber *et al.* proposed different phylogenetic relationships based on transcriptome/genome data from 20 and 32 species of lamiids, respectively, with [[[Solanales, Boraginales], Gentianales], Lamiales] [29] and [[[Solanales, Gentianales], Lamiales], Boraginales] [17] as their conclusions. Compared to previous studies, this study complements the genomic data for *Arnebia* species and has high reliability. It is based on the analysis of 130 single-copy homologous gene sets derived from high-quality genomic data of 21 representative species. In addition, by utilizing relatively complete genome and transcriptome data, along with additional transcriptome data from *L. arvense* (Flora of China) that was not previously analyzed, this study proposes a different perspective from Zhu *et al.*, POWO, and Weigend *et al.* [32–34]. Based on advanced analyses employing genomic data, we propose that the taxonomic status of *A. tschimganica* should be reverted to *Lithospermum*, which is a *Lithospermum* species related to *L. erythrorhizon*, thereby validating Johnston’s viewpoint [30]. The karyotype analysis of *A. tschimganica* indicated that it has 28 chromosomes, matching those of *L. erythrorhizon*, whereas *Arnebia* species have a 2*n* = 14 chromosome count (Fig. 1C) [35, 38]. This finding further substantiates our conclusion.

Integrative analysis from multidimensional perspectives encompassing genomics, transcriptomics, and metabolomics has emerged as a significant research approach to systematically elucidate the biosynthetic mechanisms of specific secondary metabolites [39]. In addition to investigating metabolite variations based on gene expression differences, this study also explores whether genomic-level variations are involved in regulating the biosynthesis of shikonin and its derivatives. We performed a genome-wide systematic identification of the copy number of key genes in the shikonin synthesis pathway in both *A. tschimganica* and *L. erythrorhizon*. The results revealed that *A. tschimganica* harbors 65 key genes (22 MVA pathway genes, 18 PP pathway genes, 16 *PGT*, 2 *GHQ3″H*, 3 *DSH*, and 4 *SAT/AAT*), while *L. erythrorhizon* contains 78 key genes (28 MVA pathway genes, 22 PP pathway genes, 19 *PGT*, 3 *GHQ3″H*, 2 *DSH*, and 4 *SAT/AAT*) (Table S8). The reduced copy numbers of genes in the MVA pathway genes, PP pathway genes, and *PGT* families in *A. tschimganica* compared with *L. erythrorhizon* may contribute to the lower shikonin production in its roots. Additionally, we compared the cis-acting elements in the promoters of shikonin biosynthetic enzyme genes between the two species using PlantCARE. The analysis revealed obvious differences in the types and numbers of cis-acting elements in the promoters of *LePGT1*/*2*, *LeGHQ3″H1*, *LeDSH1*, and *LeSAT*/*AAT* in *L. erythrorhizon* compared to their homologs in *A. tschimganica* (Table S12). How these differences specifically affect enzyme activity or expression regulation, thereby leading to variations in shikonin accumulation, remains to be further investigated in future studies.

Multiple TFs are known to govern metabolic synthesis of specialized phytochemicals across plant systems. In *Vitis vinifera*, the ethylene-inducible VviERF003 functions as an activator of glycosylated monoterpenoid production pathways [40]; the SmMYB36-SmERF6/SmERF115 transcriptional complex modulates tanshinones and phenolic acid biosynthesis in *Salvia miltiorrhiza* [41]; AaGSW1 can positively regulate the expression of *CYP71AV1* and *AaORA*, and significantly increase the content of artemisinin and dihydroartemisinic acid in *Artemisia annua* [42]. Research on the regulation of shikonin biosynthesis has mainly focused on three TFs (*LeERF-1*, *LeEIL-1*, and *LeMYB1*), but their target genes remain unidentified, and research progress in this area has been limited [22, 24, 43]. This study revealed that *A. tschimganica* has fewer members of ERF, bHLH, C2H2, NAC, GRAS, WRKY, and bZIP TF families compared to *L. erythrorhizon* (Table S13). These TFs are related to secondary metabolism as well as responses to environmental stresses, such as drought and low temperature [44–46], which may impact the biosynthesis of shikonin in *A. tschimganica*. Furthermore, we have reanalyzed the transcriptomic data from the roots, stems, leaves, and flowers of *A. tschimganica* and constructed the co-expression network for *AtsDSH1* (Fig. S14A). Additionally, we generated a tissue-specific expression heatmap for the candidate transcription factors identified through this co-expression network analysis (Fig. S14B). To our surprise, the transcription factors screened from the *LeDSH1* co-expression analysis did not correspond to those predicted by the *AtsDSH1* co-expression analysis, which may be attributed to the differences in the transcriptomic datasets used for the analyses. However, the regulatory roles of these transcription factors, screened based on the *AtsDSH1* co-expression network analysis, on DSH1 warrant further investigation. In addition to *DSH1*, the transcription levels of genes such as *GHQ3″H1*, *SAT1*, and *AAT2* are also significantly correlated with shikonin and its derivative contents. Various transcription factors may participate in regulating shikonin biosynthesis by modulating the expression levels of these enzyme genes. These potential regulatory mechanisms warrant systematic exploration in future work to achieve a more comprehensive understanding of the regulatory network.

Many Boraginales plants are known to contain various bioactive compounds. However, the detection of metabolites in the roots of *A. tschimganica* and *L. erythrorhizon* had previously been limited to shikonin, acetylshikonin, β,β-dimethylacrylshikonin [2], fumaric acid, caffeic acid, chlorogenic acid, and dimethyl fumarate [47]. This study is the first to systematically identify and compare the root metabolites of *A. tschimganica* and *L. erythrorhizon*. Notably, we also identified a diverse range of additional compounds in the roots of both species, including emodin, rosmarinic acid, allantoin, salvianic acid A, protocatechualdehyde, ferulic acid methyl ester, α-linolenic acid, γ-linolenic acid, and arachidonic acid (Table S11). These compounds have been shown to possess a variety of pharmacological properties, including antidiabetes, antihyperlipidemia, antifungal properties, and malaria treatment [48–50]. This discovery provides a solid theoretical basis for a comprehensive understanding and development of the medicinal and economic value of Boraginaceae plants. Furthermore, the significant upregulation of lithospermic acid B and methyl lithospermate in *A. tschimganica* points to the possibility that these compounds, which are products of branch pathways stemming from the preshikonin biosynthesis network, may compete with shikonin biosynthesis for common precursors, creating a potential competitive branch point.

## Materials and methods

### Plant materials

*Arnebia tschimganica*, *A. guttata*, and *A. szechenyi* used in this study were collected from Ili Xinjiang, Ili Xinjiang, and Alxa League Inner Mongolia, China, respectively. *Lithospermum erythrorhizon* and *L. arvense* used in this study were collected from Chifeng Inner Mongolia and Ili Xinjiang, China, respectively. *Cordia subcordata* and *H. indicum* used in this study were collected from Sanya, Hainan, China.

All plant tissues encompassing roots, stems, leaves, and flowers were snap frozen in LN₂ postharvesting and archived in ultra-low-temperature freezers (−80°C) preceding nucleic acid isolation. Environmental parameters for experimental cultivation were as follows: 26°C ± 1°C diurnal cycle (16-h light/8-h dark), photosynthetic photon flux density of 100 μmol·m^−2^·s^−1^, and humidity stabilization between 60% and 70%.

Callus induction and proliferation in *A. tschimganica* was achieved through aseptic culture on MS-based induction medium (0.4 mg/l NAA, 0.2 mg/l 2,4-D, 1 mg/l KT) at constant 26°C. The *L. erythrorhizon* callus cells were stored in our laboratory. The callus cells were subcultured in B5 medium under photoperiodic illumination, and shikonin biosynthesis was triggered through dark-phase cultivation in modified M9 medium.

### Estimation of genome size and ploidy

Genome size estimation in *A. tschimganica* was conducted through dual approaches. Cytometric quantification: fresh leaf tissue (1 cm^2^) was rapidly chopped in an MGb buffer to prepare the nuclear suspensions. Nuclei were stained with 50 μg/ml propidium iodide (15-min dark incubation), and then the PI-stained nuclei were analyzed on a flow cytometer with B73 maize reference. Survey analysis: quality-controlled 76 Gb Illumina reads were processed using Jellyfish (k-mer = 17) (http://github.com/jamesturk/jellyfish), with genome size calculated via linear regression of k-mer frequency. The ploidy of *A. tschimganica* was predicted using ploidyNGS based on the bam file of 76 Gb Illumina clean reads mapped with the *A. tschimganica* genome and performed with ggplot [51].

### Genome sequencing

Genomic DNA was extracted from leaves of an individual plant of *A. tschimganica* using a modified CTAB method [52]. The purity, concentration, and integrity of the genomic DNA were determined by Qubit 3.0 fluorometers (Thermo Fisher Scientific, USA) and Agilent 4200 Bioanalyzer, respectively. A paired-end (PE) library with an insertion size of 350 bp was constructed and sequenced on an Illumina Hiseq system.

In parallel, long-read sequencing was achieved by constructing a SMRTbell DNA library with a 15-kb insert followed by sequencing on the PacBio Sequel II system. Hi-C library preparation involved specific pretreatment steps: leaf samples cryogenically pulverized in liquid nitrogen underwent formaldehyde fixation (4% solution, room temperature) under vacuum conditions for 30 min. The crosslinking process was terminated by adding 2.5 M glycine solution with 5-min incubation, followed by 15-min ice bath treatment. Cellular pellets obtained through centrifugation (2500 rpm, 10 min, 4°C) were enzymatically digested using DpnII restriction enzyme before DNA extraction for library construction [53]. Final library amplification via PCR preceded sequencing on the Illumina NovaSeq 6000 system.

### Genome assembly and quality assessment

The 25.55 Gb HiFi reads were generated using SMRTlink v9.0.0.92188, representing about 41.7× coverage depth of the genome. *De novo* assembly of the HiFi reads was performed using hifiasm v0.14-r312 to generate primary contigs [54]. For Hi-C assembly, the 60.48 Gb Illumina clean reads were mapped to the genome draft, and the library quality was assessed by HiCUP and scaffolds were constructed by ALLHiC [55, 56]. BUSCO v5.7.1 and LAI were used to assess the integrity of the assembly [57, 58].

### Genome annotation

LTRs were identified using ltr_finder v1.05 (-D 20000 -d 1000 -L 7000 -l 100 -p 20 -C -M 0.85) [59] and ltr_harvest v1.5.1 (-similar 85 -vic 10 -seed 20 -seqids yes -minlenltr 100 -maxlenltr 20000 -mintsd 4 -maxtsd 6 -motif TGCA -motifmis 1) [60]; the results were integrated using LTR_retriever [61] and further classified using TEsorter [62]. The repetitive sequences were annotated using RepeatModeler [63] and RepeatMasker v4.0.6 [64] and further classified using DeepTE (https://github.com/LiLabAtVT/DeepTE).

Annotation of gene structure was based on three strategies: (i) cDNA sequences assembled from RNA-Seqs of root, stem, leaf, flower, and seed were compared with genomes using PASA v2.1 to predict candidate genes [65]. (ii) The protein-coding sequences of *L. erythrorhizon*, *Coffea canephora*, *S. lycopersicum*, *Olea europaea*, and *A. thaliana* were aligned with the genome, and the candidate genes were predicted by BLAST v2.2.28 and Genewise v2.2.0 [66, 67]. (iii) Augustus v3.3, GeneMark, and SNAP were used to predict candidate genes [68–70]. Finally, the protein-coding genes predicted from each method were merged by GETA v2.5.6 (https://github.com/chenlianfu/geta).

The functions of protein-coding genes were annotated by mapping sequences against UniProt, NT, NR, PFAM, eggnog, GO, and KEGG. The web addresses of all databases are provided in Table S3.

Annotation of noncoding RNAs was based on two methods: (i) The predictions of rRNA, snRNA, and miRNA were predicted by aligning the genome with Rfam (http://rfam.xfam.org/). (ii) tRNAs were identified using tRNAscan-SE [71].

### Transcriptome sequencing and assembly

RNA isolation from multiple tissues (roots, stems, leaves, flowers) of six species (*A. tschimganica*, *A. guttata*, *A. szechenyi*, *L. arvense*, *C. subcordata*, *H. indicum*) was performed using the Plant Total RNA Isolation Kit (Vazyme, #RC401, Nanjing, China). RNA quality control involved dual verification: spectrophotometric evaluation of A260/A280 ratios and electrophoretic separation on 1.0% agarose gels. The cDNA libraries were constructed for Illumina NovaSeq 6000 platform analysis. Raw data quality assessment utilized FastQC v.0.10.0 (http://www.bioinformatics.babraham.ac.uk), followed by transcript assembly via Trinity v2.4.0 with min_kmer_cov:3 [72]. Three biological replicates were conducted for each sample.

### Phylogenetic analyses

Gene family clustering among 21 species genome data: *A. tschimganica*, *L. erythrorhizon*, *E. plantagineum*, *Utricularia gibba*, *Sesamum indicum*, *Strobilanthes cusia*, *O. europaea*, *Handroanthus impetiginosus*, *Erythranthe guttata*, *Antirrhinum majus*, *S. miltiorrhiza*, *Capsicum annuum*, *Nicotiana attenuata*, *S. lycopersicum*, *Datura stramonium*, *Ipomoea nil*, *C. canephora*, *Ophiorrhiza pumila*, *Catharanthus roseus*, *Daucus carota*, *A. thaliana*, using the OrthoMCL v1.4 (http://OrthoMCL.org/OrthoMCL/) [73], and 130 single-copy orthologs were selected for phylogenetic reconstruction of lamiids. Another gene family clustering was performed on genome/transcriptome data from 14 species, including *A. tschimganica*, *L. erythrorhizon*, *E. plantagineum*, *A. euchroma*, *A. guttata*, *A. szechenyi*, *L. arvense*, *Cynoglossum officinale*, *Symphytum officinale*, *Myosotis silvatica*, *Ehretia microphylla*, *C. subcordata*, *H. indicum* and *S. lycopersicum,* using the OrthoMCL v1.4 [73]. From this analysis, 137 single-copy orthologs were selected for subsequent phylogenetic analyses of Boraginaceae species. Protein sequence alignment was performed using MUSCLE v3.8.31 (-maxiters 16) [74]. A maximum likelihood phylogenetic tree was generated using PhyML v3.0 with 1000 bootstrap replicates [74, 75]. Divergence time estimation employed the mcmctree 4.4 (model = JC69; burnin = 20 000; sampfreq = 2; nsample = 100 000) module in PAML 4.9 [76], calibrated using Timetree (http://www.timetree.org/) reference points. The expansion and contraction of gene families were quantified using CAFÉ 4.1 (-p 0.05 -r 10000) based on phylogenetic topology and clustering outputs [77]. To validate the topological structure, a phylogenetic tree was constructed using the SOI (https://github.com/zhangrengang/SOI) with the single-copy orthologs identified by OrthoFinder [36, 78]. The reference genome of *L. erythrorhizon* used for comparative analysis in this study was sourced from https://ftp2.cngb.org/pub/CNSA/data3/CNP0002039/CNS0395983/CNA0029378/. Data sources for all species genomes or transcriptomes are listed in Table S14.

### WGD and gene duplication pattern analyses

Interspecific/intraspecific collinearity patterns and gene duplication types [six categories: whole-genome duplication (WGD), tandem duplicates, proximal duplication (PD), transposed duplication (TRD), dispersed duplication (DSD), singleton] were analyzed by MCScanX [79] and DupGen_finder [80]. Synonymous substitution rates (Ks) for paralogous/orthologous gene pairs were computationally determined using wgdi [81], and Ks distribution was used to predict WGD occurrences in interspecies and intraspecific divergence events. The WGD analysis was further performed using high-confidence syntenic orthologs that were obtained by applying more stringent filtering (OI = 0.6) with the SOI (https://github.com/zhangrengang/SOI) [36].

### Widely targeted metabolome detection of plant root

Lyophilized root tissues (*A. tschimganica*/*L. erythrorhizon*) were pulverized (Retsch MM 400 ball mill). A 50-mg sample of root powder underwent cold methanol extraction (70% v/v, 1.2 ml) with vortex homogenization, followed by centrifugation (12 000 rpm, 3 min). The resulting supernatant was filtered (0.22 μm) for UPLC–MS/MS analysis. The separation of supernatant was achieved on Agilent SB-C18 columns (ExionLC™ AD system) with binary mobile phase: phase A: 0.1% formic acid/water, phase B: 0.1% formic acid/acetonitrile; gradient program: 5% B (0 min) → 95% B (9 min, linear) → 5% B (11.1 min) → equilibration (14 min total); Operational parameters: flow rate: 0.35 ml/min, column temperature: 40°C, injection volume: 2 μl. Mass spectrum conditions: ion spray voltage: +5500 V/−4500 V, electrospray ion source temperature: 550°C, gas pressures (psi): GS I 50|GS II 60|Curtain 25, collision-induced dissociation: high-energy mode. Three biological replicates were conducted for each sample. The metabolites were identified by comparing the secondary ion mass spectrometry of compounds with the Metware database, and metabolites were quantified by MRM analysis with a triple quadrupole. Peak area integration and correction of chromatographic peaks for all metabolites were performed using MultiQuant [82]. Metabolite difference analysis was performed using OPLS-DA (orthogonal partial least squares-discriminant analysis). VIP (variable importance in projection) >1, fold change ≥2, and fold change ≤0.5 were established as statistically significant discriminators of interspecies metabolic divergence.

### Cross-species comparative transcriptome analysis

Transcriptomic datasets of *L. erythrorhizon* (whole roots, leaves, and light-/dark-cultured hairy roots in B5/M9 media) were acquired from NCBI SRA (SRP141330, SAMN13650849, SAMN13650867) for gene expression analysis. Read alignment quantification was performed using featureCounts (1.5.0-p3), followed by the transcripts per kilobase of the exon model per million (TPM) for each gene using Kallisto [83]. DESeq2 v.1.20.0 was employed to analyze the differential expression of genes in different tissues of *A. tschimganica* or *L. erythrorhizon* [84]. The optimal matching gene pairs of *A. tschimganica* and *L. erythrorhizon* were obtained by reciprocal BLAST method. The TPM values of the optimal matching gene pairs were sorted into the expression matrix, and then quantile normalization was performed using the R package preprocessCore v1.44.0, and cross-species comparative transcriptome analysis was performed [85]. Transcript abundance was standardized through log2 (TPM + 1) transformation, and a clustering heatmap was drawn using the R package pheatmap.

### RNA extraction and RT-qPCR analysis

RNA isolation from *A. tschimganica* and *L. erythrorhizon* tissues was performed via the Plant Total RNA Isolation Kit. The cDNA synthesis was achieved using HiScript II 1st Strand cDNA Synthesis Kit (Vazyme, #R212), and qPCR was performed using the AceQ Universal SYBR qPCR Master Mix (Vazyme, #Q511) on StepOnePlus™ Real-Time PCR System. Gene expression values were calibrated against *GAPDH* (endogenous reference gene) and calculated through the 2^−ΔΔCt^ method [86]. Experimental reproducibility was ensured by triplicate independent biological replicates (primer details: Table S15).

### Heterologous expression of the *AtsDSH1* and substrate-feeding experiments

The coding sequence (CDS) of *AtsDSH1* was amplified using gene-specific primers and subsequently cloned into the pYES2 vector (digested with BamHI/BamHI) via the ClonExpress Ultra One Step Cloning Kit V2 (C116, Vazyme). The recombinant plasmid pYES2::AtsDSH1 and the empty vector pYES2 were separately transformed into competent cells of *S. cerevisiae* strain WAT11 using the lithium acetate method. The transformed cells were plated on SD-Ura medium and incubated at 28°C for 3–5 days. Single colonies were selected and inoculated into 7.5 ml of SD-Ura liquid medium, followed by incubation at 28°C with shaking at 230 rpm until the OD600 reached 1.2–1.4. Yeast cells were collected by centrifugation at 1000 × *g* for 10 min and washed twice with 20 ml of ddH₂O. The cells were then resuspended in 7.5 ml of SC-Ura liquid medium and cultured at 28°C and 220 rpm for 5 h. Subsequently, 2% galactose and 1% raffinose were added to the SC-Ura medium, and cultivation continued at 28°C and 220 rpm for 20 h. Afterward, 2 mg of the substrate deoxyshikonin was supplemented into 7.5 ml of the engineered yeast culture, which was further shaken at 28°C and 220 rpm for 48 h. The yeast cells were harvested by centrifugation at 13 800 × *g* for 10 min and subjected to ultrasonic-assisted extraction twice with 5 ml of methanol. The mixed methanol extracts were evaporated using a rotary evaporator. The residue was finally redissolved in 500 μl of methanol for HPLC analysis. All primer sequences are provided in Table S15.

HPLC analysis was conducted on an Agilent 1260 system coupled with an Agilent Infinity UV detector. Chromatographic separation was achieved using a mobile phase comprising 0.1% trifluoroacetic acid in water (A) and 100% acetonitrile (B) (30:70, v/v), at a constant flow rate of 1.0 ml/min. The injection volume was 10 μl. The analytes were detected at a wavelength of 515 nm. Deoxyshikonin and shikonin standards were purchased from Shanghai Yuanye Bio-Technology Co., Ltd, China.

### Co-expression network analysis

Transcriptomic mining of *L. erythrorhizon* datasets revealed regulatory candidates for *LeDSH1* through integrative evaluation of gene expression and co-expression network assessments. The raw data (.CEL files) were preprocessed and normalized through RMA normalization using the R package affy. Genes with expression values below or equal to the thresholds (0, 0.5, 1) in all samples were removed by filtering progressively, refining the transcriptome landscape to <45 000 high-confidence genes. The gene co-expression matrix was constructed and modularized using the R package WGCNA [87]. Then genes within the module of *LeDSH1* and those with a correlation greater than 0 were selected, and the ITAK was used to identify genes predicted to be transcription factors among them [88]. Subsequently, transcription factors with correlation values (weight values) below 0.1 were excluded, and the interaction network between *LeDSH1* and transcription factor genes with strong correlation was visualized using Cytoscape v3.7.1 [89]. Using the same approach as described above, a co-expression network analysis of *AtsDSH1* and screening of candidate transcription factors were conducted based on the transcriptome data of *A. tschimganica* tissues.

### Analysis of cis-regulatory elements and Y1H assay

The cis-regulatory elements of the promoter region (2000 bp sequence upstream of the gene’s 5′UTR) of biosynthetic enzyme genes for shikonin and its derivatives were analyzed using the PlantCARE (https://bioinformatics.psb. ugent.be/webtools/plantcare/html/). The cis-regulatory elements in the promoter regions of *LeDSH1* and At14g1402.1 were further analyzed using JASPAR (https://jaspar.elixir.no/). The full-length coding sequences (CDSs) of LE12272.1, LE12347.1, At04g0834.1, and At01g2338.1 were amplified and ligated separately in the pGADT7 vector. Promoter regions of *LeDSH1* and At14g1402.1 were inserted into the pHIS2 vector. The pGADT7-GUS (β-glucuronidase) vector served as negative control. Competent Y187 cells were cotransfected with paired plasmids (pGADT7-TF/pHIS2-promoter) using the lithium acetate method. The transformants were cultivated on SD/-Trp/-Leu/-His. To identify the interactions between transcription factors with promoters, the positive colonies were inoculated on 75 mM 3-AT supplemented medium (SD/-Trp/-Leu/-His) and grown for 3–5 days at 28°C (primer details: Table S15).

### Dual-luciferase reporter assay

Transcriptional activity quantification was performed using the dual-luciferase reporter system following validated protocols [90]. Four candidate regulators (LE12272.1, LE12347.1, At04g0834.1, and At01g2338.1) were engineered into effector vectors (pGreenII 62-SK). Corresponding promoter regions (*LeDSH1*, At14g1402.1) were cloned into pGreenII 0800 reporter constructs. Empty effector vector served as negative control. All recombinant vectors were transformed into *Agrobacterium tumefaciens* GV3101(pSoup-P19) competent cells. Different combinations of effector and reporter were mixed in a 1:1 ratio and transiently co-transformed into *Nicotiana benthamiana* leaves. The LUC-to-REN activity was observed using the Tanon 5200 Chemiluminescent Imaging System. Experimental validity was confirmed through triplicate biological replicates (primer details: Table S15).

## Supplementary Material

Web_Material_uhag077

## Data Availability

The genome assembly of *A. tschimganica* and corresponding multi-omics datasets (WGS and transcriptome sequencing in this study) are publicly accessible through the NCBI BioProject repository (Accession: PRJNA1209689). Additional supporting datasets, including analytical results and supplementary evidence, are comprehensively documented in the main manuscript and associated supplementary materials.
