# Supplementary material for: Genomics meets metabolomics: decoding Arnebia tschimganica and the shikonin biosynthesis pathway
Source: Hortic Res. 2026 Mar 3;13(6):uhag077. doi: 10.1093/hr/uhag077 (PMC13253341; doi:10.1093/hr/uhag077)
Supplement: Web_Material_uhag077 [file web_material_uhag077.zip › Supplementary Figures-20260110_Clean.docx]

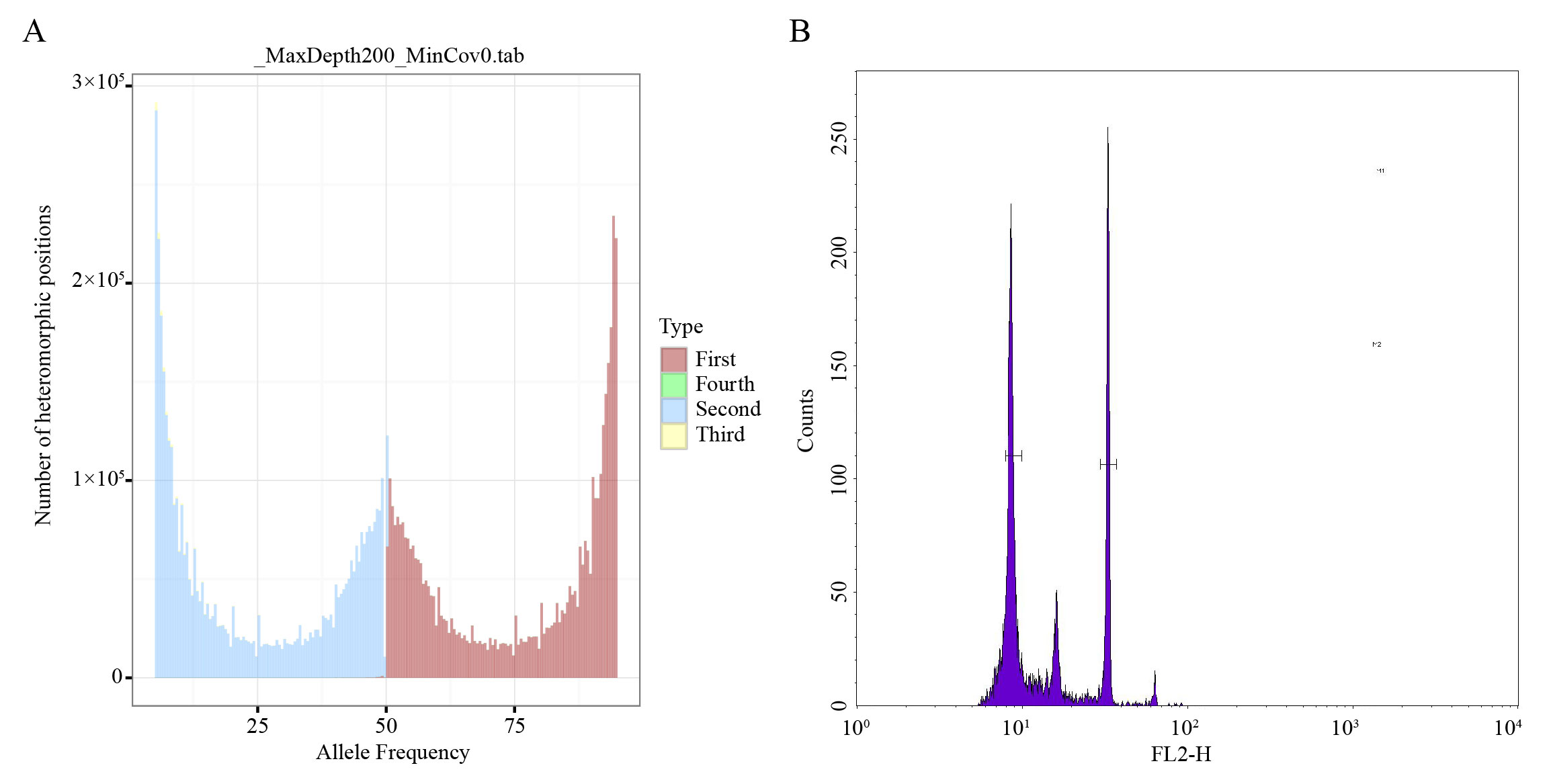


Supplementary Figure 1. Results of ploidy (A) and genome size (B) prediction using ploidyNGS and flow cytometry, respectively, for *A. tschimganica*.


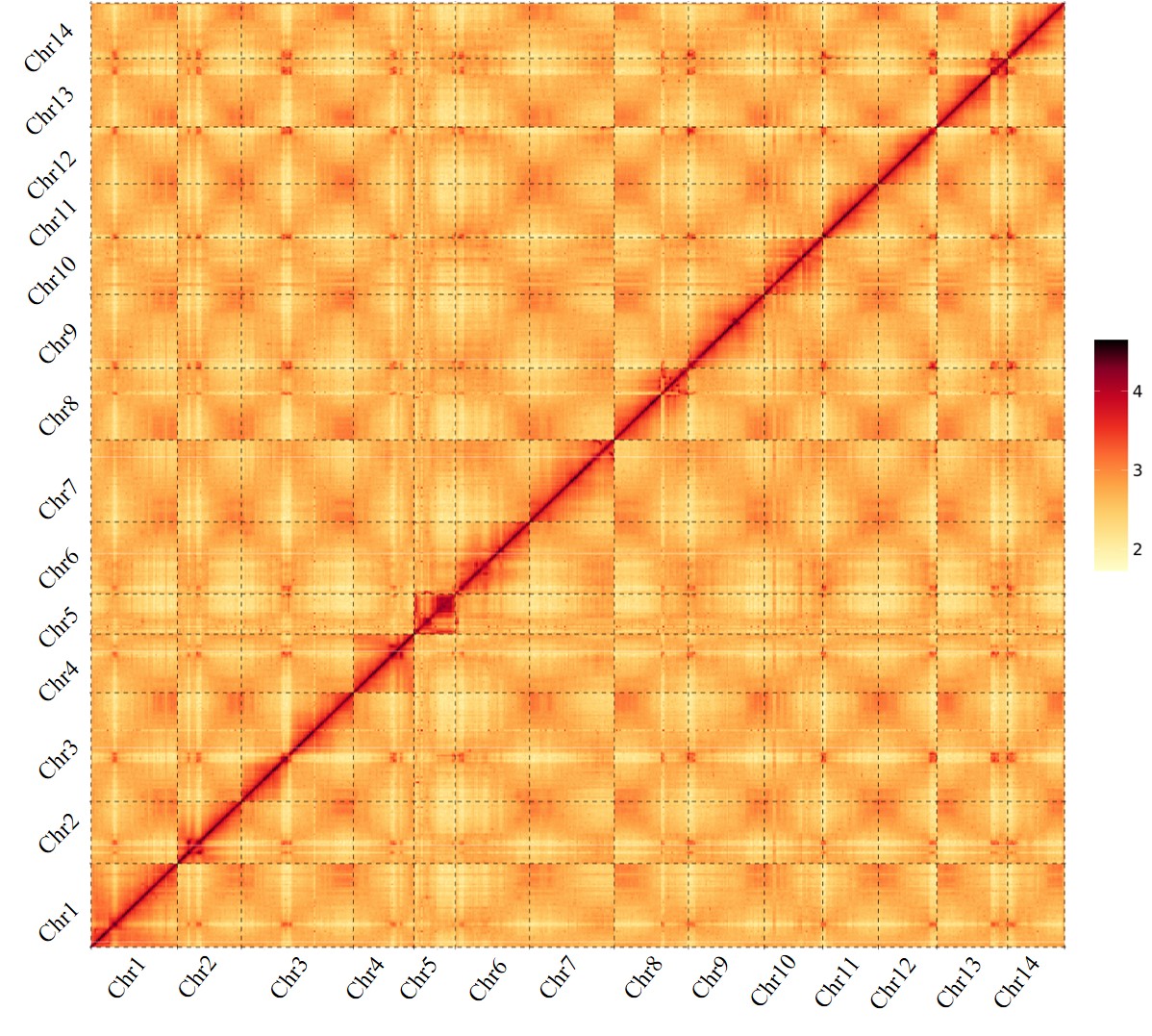


Supplementary Figure 2. Hi-C contact map of chromosome-scale genome assembly of *A. tschimganica*.


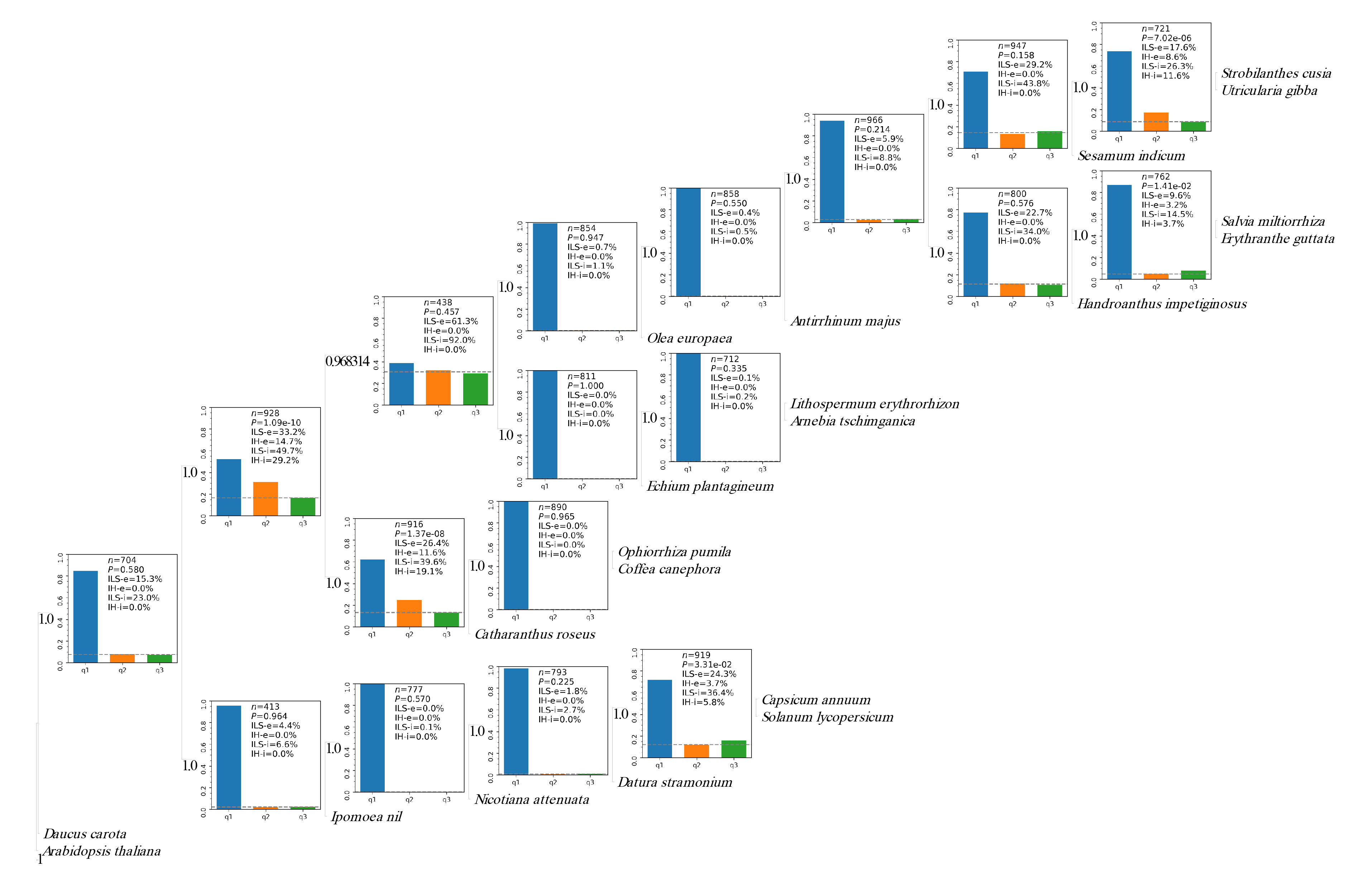


Supplementary Figure 3. The phylogenetic tree of Boraginales, Gentianales, Solanales and Lamiales was constructed using the single-copy orthologs from the syntenic orthogroups that were inferred with an OI of 0.6 in SOI.


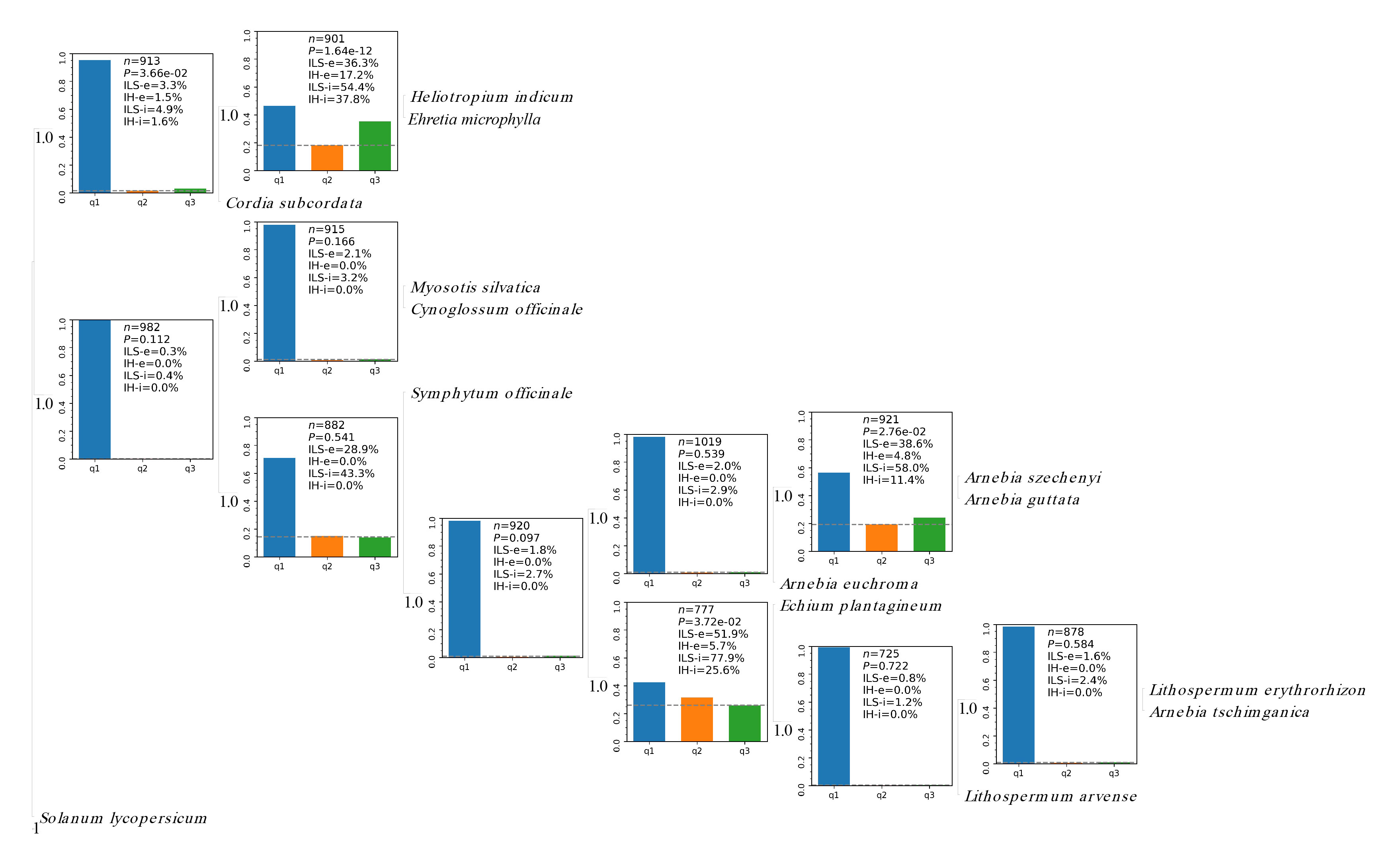


Supplementary Figure 4. The phylogenetic tree of 13 Boraginaceae species and *S. lycopersicum* was constructed using the single-copy orthologs from the syntenic orthogroups that were inferred with an OI of 0.6 in SOI.


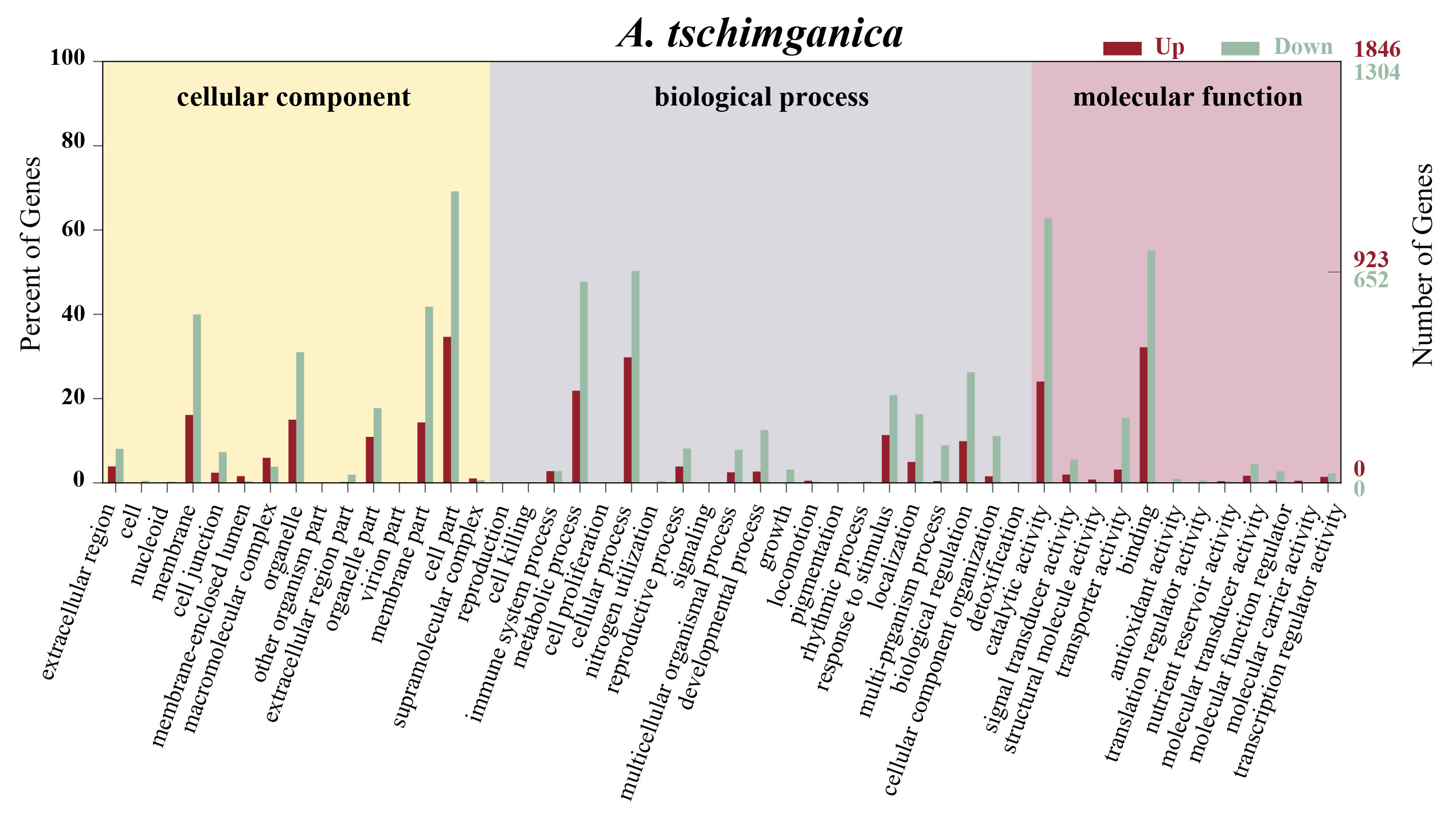


Supplementary Figure 5. Gene Ontology term enrichment analyses of the 441 expanded and 2356 contracted gene families of *A. tschimganica*.


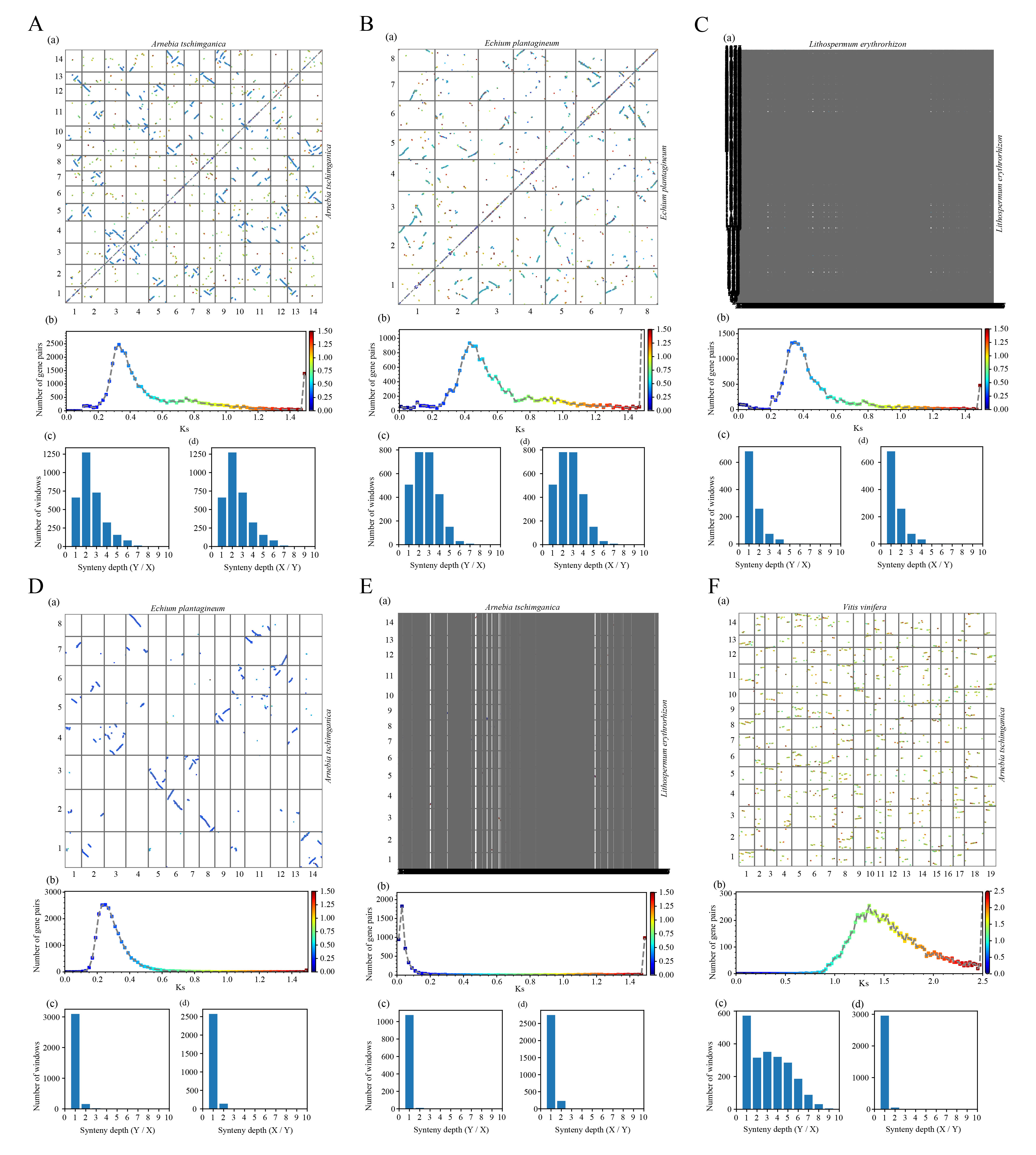


Supplementary Figure 6. Ks-colored dot plots of synteny after applying an OI of 0.6.

A. Ks-colored dot plot of syntenic regions within the *A. tschimganica* after applying an OI of 0.6. B. Ks-colored dot plot of syntenic regions within the *E. plantagineum* after applying an OI of 0.6. C. Ks-colored dot plot of syntenic regions within the *L. erythrorhizon* (contig-level genome) after applying an OI of 0.6. D. Ks-colored dot plot of syntenic regions between *A. tschimganica* and *E. plantagineum* after applying an OI of 0.6. E. Ks-colored dot plot of syntenic regions between *A. tschimganica* and *L. erythrorhizon* (contig-level genome) after applying an OI of 0.6. F. Ks-colored dot plot of syntenic regions between *A. tschimganica* and *V. vinifera* after applying an OI of 0.6. (a) dot plots with colored by Ks (x-axis and y-axis, chromosomes of the two genomes; a dot indicates a homologous gene pair between the two genomes); (b) histogram of Ks; (c-d) synteny depth derived from 50-gene windows.


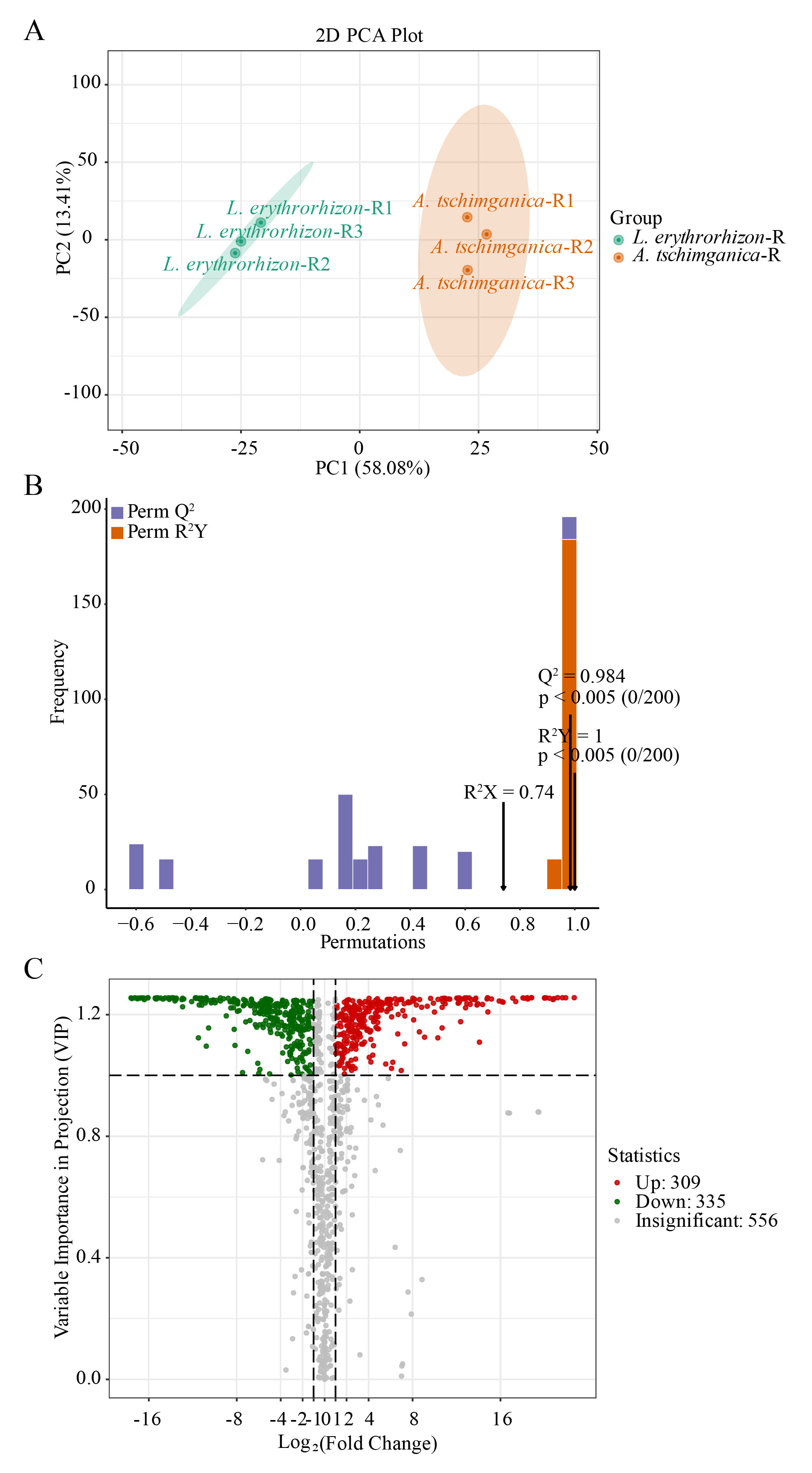


Supplementary Figure 7. Principal component analysis diagram (A) and OPLS-DA validation diagram (B) of the metabolites of *A. tschimganica* and *L. erythrorhizon* roots, and volcanic map of differential metabolites (C) of *A. tschimganica* and *L. erythrorhizon* roots screened based on Log_2_FC and VIP.


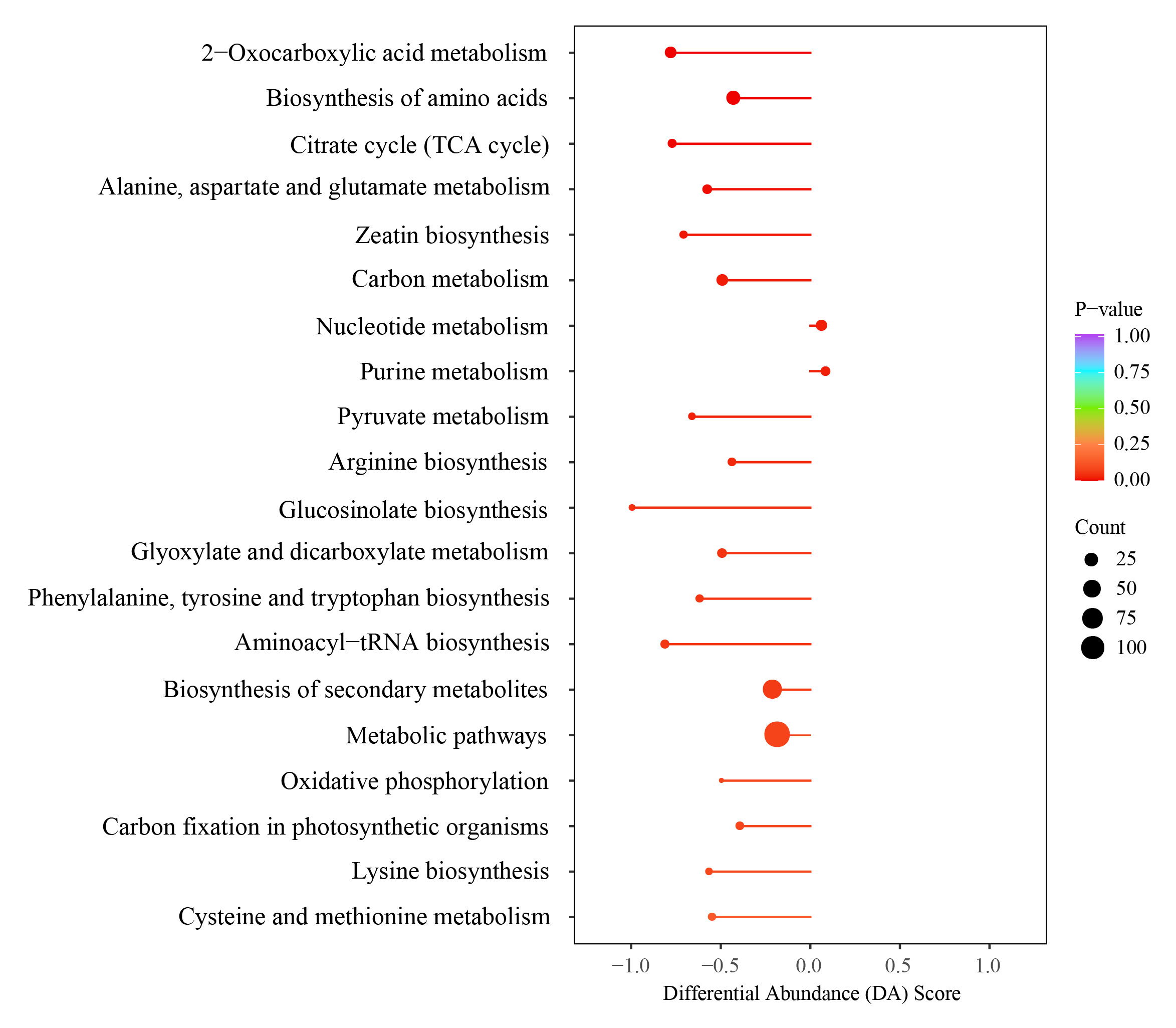


Supplementary Figure 8. Differential abundance scores of differential metabolites in the roots of *A. tschimganica* and *L. erythrorhizon*.


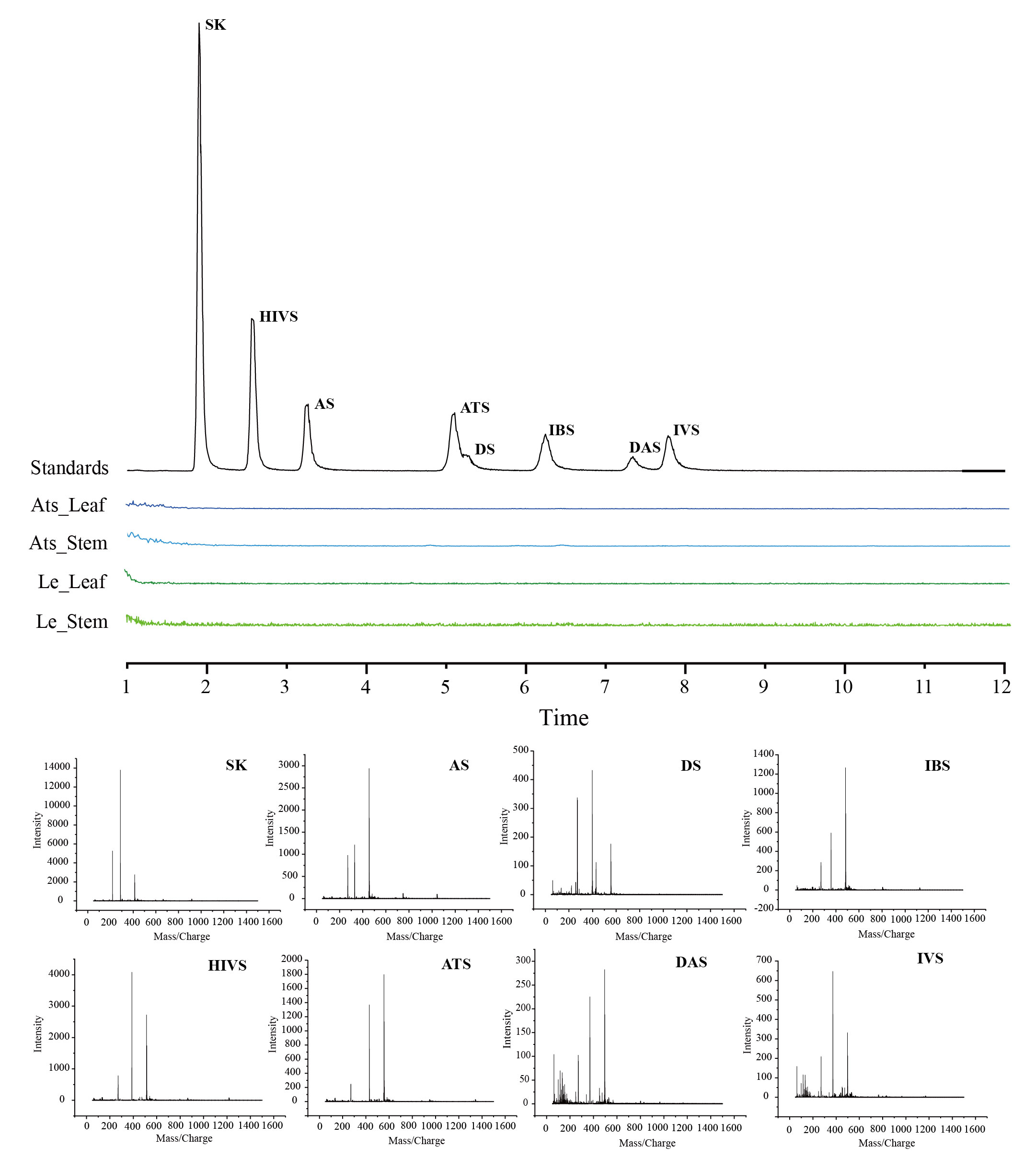


Supplementary Figure 9. LC-MS analysis result of deoxyshikonin, shikonin, and six derivatives in the leaves and stems of *A. tschimganica* and *L. erythrorhizon*. SK: Shikonin, HIVS: β-Hydroxyisovalerylshikonin,AS: Acetylshikonin, ATS: β-acetoxyisovalerylalkannin, DS: Deoxyshikonin, IBS: Isobutylshikonin, DAS: β,β-Dimethylacrylalkannin, IVS: Isovalerylshikonin.


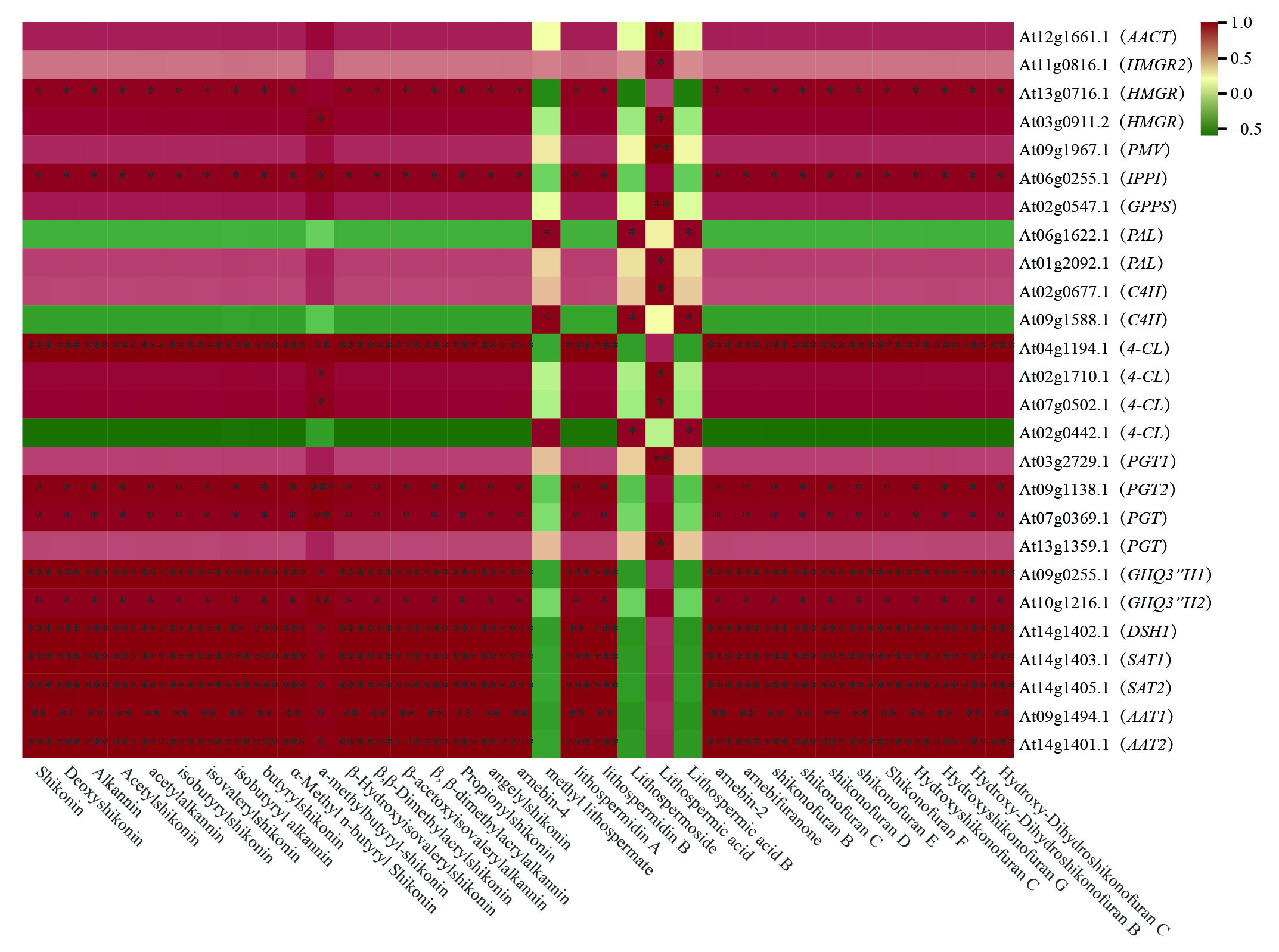


Supplementary Figure 10. The correlation heatmap of gene expression levels of shikonin biosynthetic enzymes versus shikonin and its derivatives contents.


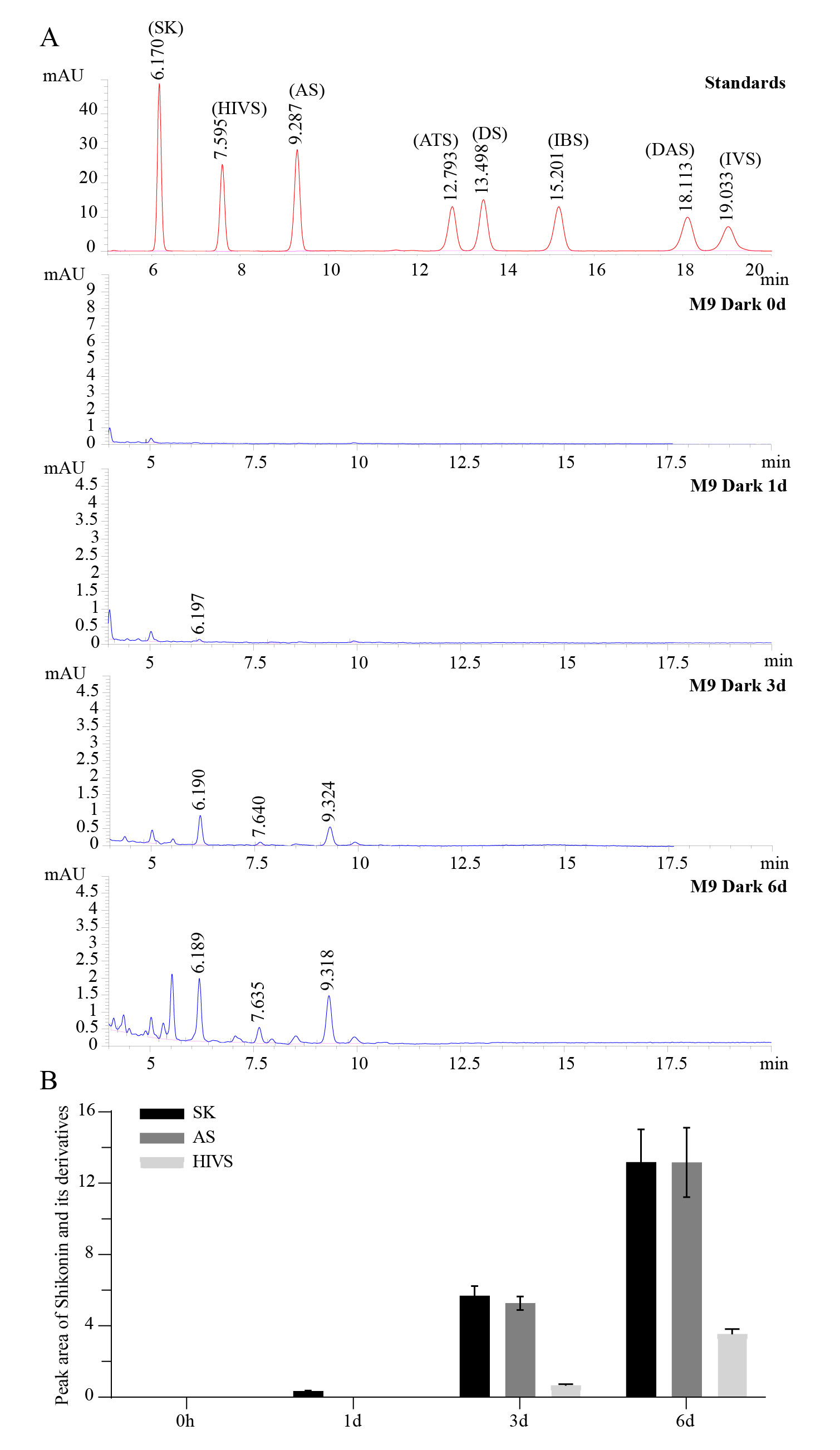


Supplementary Figure 11. HPLC of shikonin and its derivatives content (A) and statistical of peak area of shikonin and its derivatives (B) secreted from the callus of *A. tschimganica* in dark culture in M9 medium at 0 h、1 d、3 d、6 d. SK: Shikonin, HIVS: β-Hydroxyisovalerylshikonin,AS: Acetylshikonin, ATS: β-acetoxyisovalerylalkannin, DS: Deoxyshikonin, IBS: Isobutylshikonin, DAS: β,β-Dimethylacrylalkannin, IVS: Isovalerylshikonin.


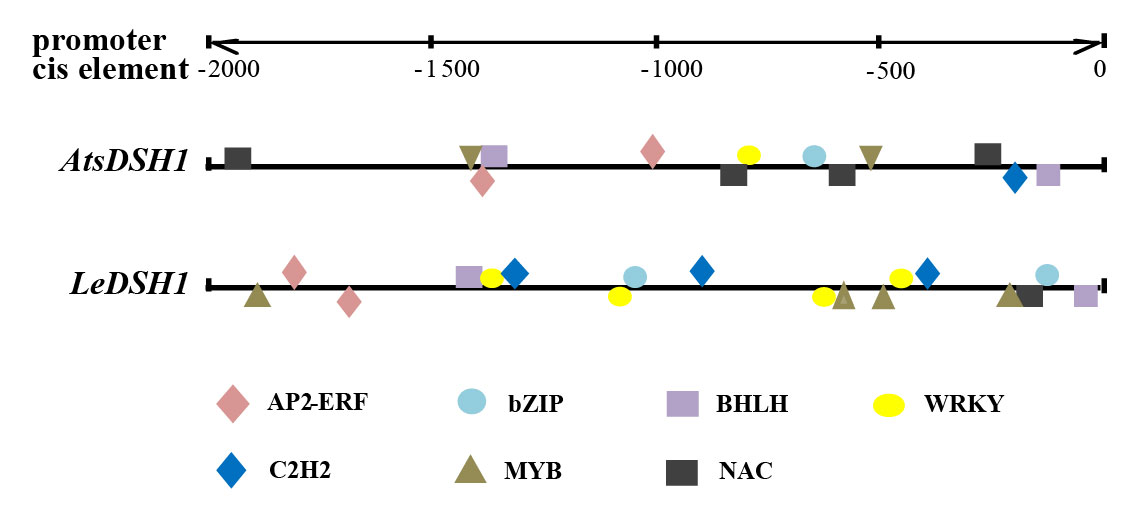


Supplementary Figure 12. Some of the cis-acting elements in the promoters of *LeDSH1* and its homologous gene *AtsDSH1* were analyzed using JASPAR (https://jaspar.elixir.no/).


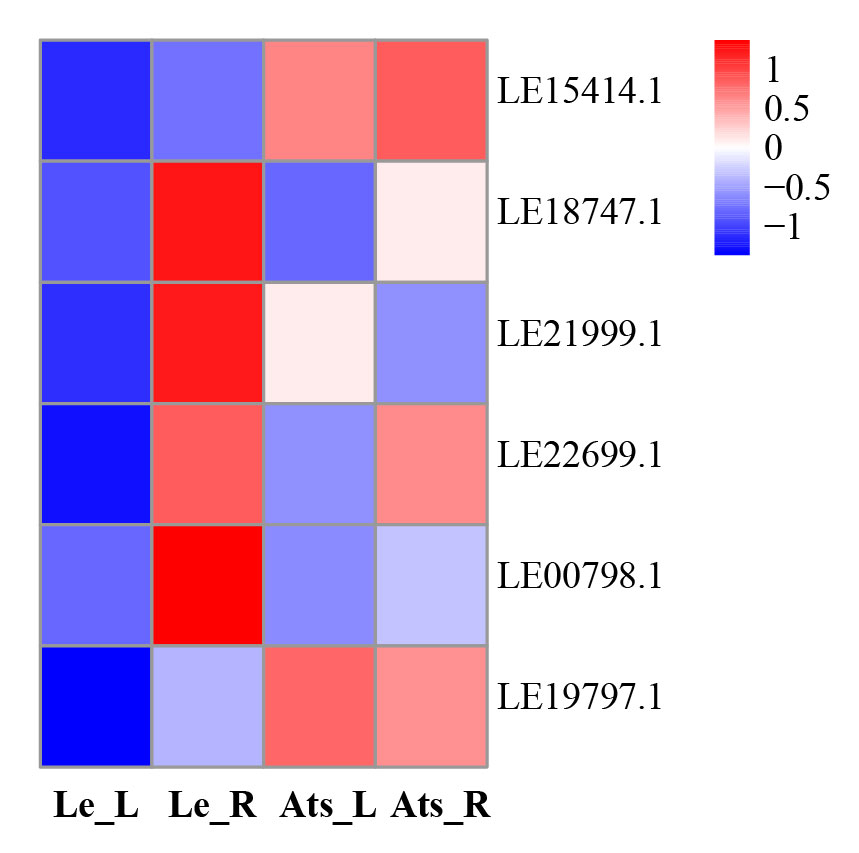


Supplementary Figure 13. Heatmap depicting expression patterns of transcription factors identified through cross-species comparative transcriptomic analysis, showing root-specific upregulation in *L. erythrorhizon* relative to leaves but non-differential expression between root and leaf tissues in *A. tschimganica*.


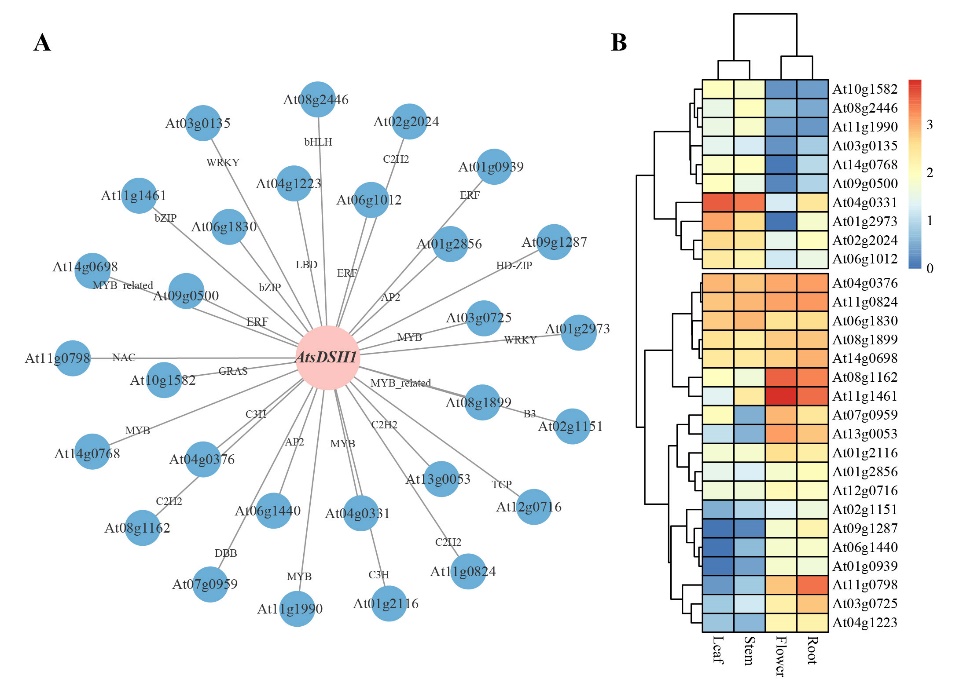


Supplementary Figure 14. The candidate transcription factors of *AtsDSH1* were screened through the co-expression network analysis (A) and heat map of tissue expression patterns of candidate transcription factors (B).
